# Comparative Diagnostic Accuracy of the American College of Radiology Thyroid Imaging Reporting and Data System (ACR TI-RADS) and the European Thyroid Imaging and Reporting Data System (EU-TIRADS) for Thyroid Nodule Malignancy: A Systematic Review

**DOI:** 10.7759/cureus.119522

**Published:** 2026-11-11

**Authors:** Shane Buckley, Amro M Karoratna, Bethany Cushing

**Affiliations:** 1 Radiology, St Michael’s Hospital, Dublin, IRL; 2 Emergency, St Michael's Hospital, Dublin, IRL; 3 General Practice, St Michael’s Hospital, Dublin, IRL

**Keywords:** acr ti-rads, diagnostic accuracy, eu-tirads, thyroid cancer, thyroid nodules, ultrasonography

## Abstract

Background: The American College of Radiology Thyroid Imaging Reporting and Data System (ACR TI-RADS) and European Thyroid Imaging Reporting and Data System (EU-TIRADS) estimate thyroid nodule malignancy risk but use different category definitions and fine-needle aspiration thresholds. This diagnostic test accuracy (DTA) systematic review compared their performance, focusing on ACR TR5 and EU-TIRADS 5.

Methods: PubMed, Europe PMC, Semantic Scholar, OpenAlex, Google Scholar, and Crossref were searched through October 12, 2026. Adult studies reporting diagnostic findings for either system were assessed. All three authors participated in selection and extraction; disagreements were resolved by discussion. Risk of bias was assessed using the Quality Assessment of Diagnostic Accuracy Studies 2 (QUADAS-2) tool and certainty with the Grading of Recommendations Assessment, Development, and Evaluation approach for DTA studies (GRADE-DTA). Findings were synthesized narratively. The review was not prospectively registered; no public protocol was available.

Results: Twenty-five reports representing 24 cohorts were included. Two studies provided verified 2×2 data for the highest-risk-category comparison (2,786 nodules; 2,742 patients). In Wang et al., ACR versus EU-TIRADS sensitivity was 86.1% versus 86.3%, and specificity was 90.3% versus 85.8%. In Shen et al., sensitivity was 88.2% versus 93.3%, and specificity was 87.5% versus 80.9%. QUADAS-2 identified important risk-of-bias concerns in two studies. GRADE-DTA certainty was moderate for ACR sensitivity and specificity and EU-TIRADS specificity, and low for EU-TIRADS sensitivity. Pooling and formal paired comparison were not possible.

Conclusion: EU-TIRADS was more sensitive and ACR TI-RADS more specific in these two studies, but the limited evidence and absence of paired discordant classifications preclude claims of superiority.

## Introduction and background

Thyroid nodules are common in clinical practice, and the discovery of such nodules has increased due to the routine use of ultrasonography for diagnosis. Though the majority of thyroid nodules are non-malignant, identification of those that are associated with malignancy is crucial to decide on investigation and treatment. Ultrasonography is used for assessing nodule morphology, estimation of the malignancy risk, and making a decision about fine-needle aspiration biopsy (FNA) or imaging follow-up [1,2]. Nevertheless, different approaches to sonographic features' interpretation and choosing nodules for biopsy might account for the variance in practice; hence, the need for a risk stratification system has emerged.

The American College of Radiology Thyroid Imaging Reporting and Data System (ACR TI-RADS) is a point-based system. It assigns points across five ultrasound feature groups - composition, echogenicity, shape, margin, and echogenic foci - and sums the points to classify a nodule as TR1 (benign), TR2 (not suspicious), TR3 (mildly suspicious), TR4 (moderately suspicious), or TR5 (highly suspicious). A total of 7 or more points is required for TR5. Thus, TR5 represents a cumulative score threshold that can be reached through different combinations of suspicious ultrasound features. The total category is not itself an FNA recommendation: ACR size thresholds are applied separately. In general, FNA is recommended at ≥2.5 cm for TR3, ≥1.5 cm for TR4, and ≥1.0 cm for TR5; TR1 and TR2 do not meet ACR FNA criteria [3].

The European Thyroid Imaging Reporting and Data System (EU-TIRADS) is a pattern-based system for adult thyroid nodules. It classifies nodules as EU-TIRADS 2 (benign), 3 (low risk), 4 (intermediate risk), or 5 (high risk). EU-TIRADS 5 is assigned when at least one high-suspicion feature is present, including a non-oval shape, irregular margins, microcalcifications, or marked hypoechogenicity. Unlike ACR TIRADS TR5, EU-TIRADS 5 is not defined by a cumulative point total; a single qualifying high-risk feature can be sufficient. As with ACR TI-RADS, the category and the FNA recommendation are distinct: the guideline generally recommends FNA at ≥20 mm for EU-TIRADS 3, ≥15 mm for EU-TIRADS 4, and ≥10 mm for EU-TIRADS 5; EU-TIRADS 2 generally does not require FNA [4].

Although ACR TI-RADS and EU-TIRADS share the same goal (uniform risk estimation), their highest-risk categories are not constructed using equivalent rules. ACR TR5 requires a cumulative score of at least 7 points across five ultrasound feature groups, whereas EU-TIRADS 5 is assigned when one or more specified high-suspicion features are present. Comparing these categories therefore evaluates the highest-risk category in each system, not identical ultrasound criteria or necessarily identical underlying malignancy-risk thresholds. The systems also differ in the size thresholds used to recommend FNA. Consequently, a nodule may receive different risk categories or management recommendations under the two systems. A comparison of highest-risk categories therefore addresses a different question from a comparison of guideline-based FNA recommendations.

Previous head-to-head studies have compared ACR TI-RADS and EU-TIRADS alongside other classification systems, including studies of adults referred for ultrasound-guided FNA and studies restricted to small nodules [5-7]. These reports provide useful context, but their populations and endpoints are not necessarily equivalent to a direct comparison of the two category-5 thresholds. In particular, FNA recommendation incorporates nodule size and management rules, whereas category-based diagnostic accuracy evaluates a fixed classification threshold.

Previous systematic reviews have reported variation in diagnostic performance across ultrasound risk-stratification systems and thresholds. Additional 2023 systematic reviews broadened this context: Kim et al. compared six ultrasound risk-stratification systems across 39 studies and 49,661 patients [8]; Yang et al. compared four systems across 88 studies and 59,304 nodules [9]; and Joo et al. synthesized diagnostic performance across ultrasound-based risk-stratification systems [10].

An important unresolved issue is whether the two systems differ when applied to the same adult nodules using equivalent category thresholds, rather than comparing non-equivalent FNA decisions or results from different populations. This review therefore evaluated the diagnostic performance of ACR TI-RADS and EU-TIRADS in adults, emphasizing the matched category-5 comparison where verifiable study-level 2×2 data were available.

For clarity, sensitivity is the proportion of malignant nodules classified as test-positive, whereas specificity is the proportion of benign nodules classified as test-negative. Positive predictive value (PPV) is the probability of malignancy after a positive result, and negative predictive value (NPV) is the probability of benign disease after a negative result; both depend on disease prevalence. The diagnostic odds ratio (DOR) summarizes the ratio of the odds of a positive result in malignant versus benign nodules. The area under the receiver operating characteristic curve (AUC) summarizes discrimination across possible thresholds. These measures describe diagnostic classification and should not be conflated with the accuracy of a size-dependent FNA recommendation.

The primary objective of the current systematic review was to compare the accuracy of ACR TI-RADS with that of EU-TIRADS in distinguishing between benign and malignant thyroid nodules in adults. The additional objectives included the comparison of the sensitivity, specificity, PPV, and NPV of both diagnostic systems when they are provided in the selected papers; the evaluation of the diagnostic performance of these systems at equivalent risk strata and classification criteria; the assessment of the methodological quality and bias of the included studies; and the identification of possible sources of heterogeneity in the data.

## Review

Methods

Review Strategy

This systematic review was structured using the Population, Intervention, Comparator, Outcome, and Time (PICOT) framework and reported with reference to the Preferred Reporting Items for Systematic Reviews and Meta-Analyses (PRISMA) 2020 and relevant diagnostic test accuracy (DTA) reporting considerations. The completed PRISMA-DTA reporting checklist is provided in Appendix E. The review was not registered in the International Prospective Register of Systematic Reviews (PROSPERO), and no publicly accessible prospective protocol was identified. PubMed, Europe PMC, Semantic Scholar, OpenAlex, Google Scholar, and Crossref were searched through October 12, 2026, without publication-year restrictions. Embase, Scopus, Web of Science, and the Cochrane Library were not searched because access to these databases was unavailable.

The search concepts covered thyroid nodules and lesions, ACR TI-RADS, EU-TIRADS, related TI-RADS terminology, direct comparisons between the systems, and diagnostic performance or malignancy. The source-specific search strategies and the level of detail recoverable from the archived search records are provided in Appendix F.

For Semantic Scholar and Google Scholar, four separate searches were documented; the first three query wordings and a description of the fourth are reproduced in Appendix F. OpenAlex and Crossref were each searched using four separately documented search queries through Publish or Perish (PoP), which are also reproduced in Appendix F. The review's search end date was October 12, 2026, but source-specific execution timestamps and complete PoP settings were not retained. Accordingly, Appendix F distinguishes verbatim archived strings from query descriptions and explicitly identifies the unavailable execution metadata. Embase, Scopus, Web of Science, and the Cochrane Library were not searched because access was unavailable.

Review Question and Eligibility Framework

This systematic review evaluated the comparative diagnostic performance of the ACR TI-RADS and the EU-TIRADS for identifying malignancy in thyroid nodules.

The primary comparative analysis concerned the highest-risk category in each system (ACR TR5 versus EU-TIRADS 5) when exact study-level 2×2 data were available. This is described as a matched category-5 comparison for reporting convenience, but the category definitions are not identical: ACR TR5 is a cumulative score of ≥7 points, whereas EU-TIRADS 5 requires at least one specified high-suspicion feature. The comparison is therefore a comparison of the respective highest-risk categories, not a claim that the underlying feature thresholds are equivalent. FNA-recommendation outcomes and other non-equivalent diagnostic constructs were analyzed separately rather than treated as directly interchangeable. The operational category definitions and separate FNA size thresholds are summarized in Appendix D.

Studies were eligible for inclusion when they evaluated thyroid nodules in an adult population (participants aged 18 years or older), assessed ACR TI-RADS and/or EU-TIRADS, and reported a diagnostic finding relevant to distinguishing benign from malignant nodules. Studies evaluating only one of the two systems were eligible for the overall descriptive review; a direct head-to-head comparison was not required for inclusion. The eligible diagnostic constructs included fixed category thresholds, guideline-based FNA recommendations incorporating nodule size, and other explicitly defined category or risk-stratification comparisons. Prospective and retrospective clinical diagnostic-performance studies were retained; no design-based restriction between these two designs was applied in the available selection record. The included reports used histopathology, cytology/FNA, mixed cytological and histopathological assessment, or clinical/imaging follow-up as reference standards. These reference standards were recorded as reported rather than treated as interchangeable; differences in verification and reference-standard methods were considered in the risk-of-bias and narrative interpretation. Studies or subgroups using selected surgical/biopsy populations, modified thresholds, computer-aided diagnosis (CAD), cytology-specific strata, or FNA-recommendation endpoints could contribute to the descriptive synthesis when relevant, but were not assumed to estimate the same diagnostic construct as routine-reader, fixed category-5 classification. The primary matched comparison was restricted to ACR TR5 versus EU-TIRADS 5 data with verified exact 2×2 cells. A report involving a mixed-age population was excluded at final eligibility reconciliation when results for adults could not be separated from results for participants younger than 18 years. Reports with overlapping cohorts were retained as companion publications where relevant but were not counted as independent cohorts. The operational eligibility criteria and the handling of different study types, diagnostic endpoints, and overlapping reports are summarized in Appendix G.

Data Extraction

All authors participated in the study-selection process. After records were imported into Zotero (Corporation for Digital Scholarship, Vienna, VA, USA) and duplicates were removed, the authors screened titles and abstracts for relevance. Potentially eligible reports were then retrieved in full text and assessed against the review's documented eligibility criteria.

All authors also participated in data extraction using a predesigned Microsoft Excel form (Microsoft Corp., Redmond, WA, USA). Disagreements arising during study selection or data extraction were resolved through discussion until consensus was reached. Extracted information included study characteristics, population and nodule characteristics, ultrasound classification systems and thresholds, reference standards, and diagnostic accuracy data where available.

Extracted items included study identity and publication year, setting, population and sample size, age or adult-eligibility information, reference standard, the TI-RADS systems and thresholds evaluated, diagnostic endpoint, and the availability of data for diagnostic-accuracy calculations. Where available, true-positive (TP), false-positive (FP), false-negative (FN), and true-negative (TN) counts were recorded separately for ACR TI-RADS and EU-TIRADS. Distinct analytic populations, thresholds, and endpoints reported within the same publication were kept separate. Exact integer 2×2 cells were not inferred from rounded sensitivity or specificity estimates. When a source reported the relevant TP and FP counts together with the total numbers of malignant and benign nodules, the complementary FN and TN counts were derived by subtraction from those source-reported totals, as described for Shen et al. below. Source-level discrepancies in denominators or reported accuracy were retained as limitations and documented rather than silently harmonized.

No study authors were contacted to obtain missing diagnostic data or clarification. The study-level extraction and Quality Assessment of Diagnostic Accuracy Studies 2 (QUADAS-2) mapping were checked against the final 24-cohort set (25 reports, including one companion report) and the original articles. Calculations based on available integer 2×2 cells were checked against the source reports and the study-level verification workbook.

The eight reports excluded at full-text assessment or final eligibility reconciliation, with their recorded reasons, are listed in Appendix A. Seven were excluded at the initial full-text stage; one additional report was excluded during final reconciliation because the adult subgroup could not be separated. The reasons for the eight full-text exclusions are detailed in Appendix A.

Study Selection

Initially, 5,590 records were identified from all the databases (PubMed 742, Europe PMC 1,412, Semantic Scholar 224, Google Scholar 55, OpenAlex 374, and CrossRef 2,783). A total of 1,457 duplicates were found. After removal of duplicates, 4,133 records remained. These 4,133 records were initially screened (at the title and abstract level). Most of them were excluded as not relevant to the inclusion criteria (4,097 studies). Subsequently, 36 full-text articles and trial records were eligible for full-text screening.

The selection flow is reported at the record and report levels. Six sources yielded 5,590 records; 1,457 cross-database duplicates were removed, leaving 4,133 records for title-and-abstract screening. Of these, 4,097 were excluded, and 36 reports were sought for retrieval. Three reports were not retrieved, and 33 full-text reports were assessed. Seven reports were excluded at the initial full-text assessment, leaving 26 provisionally retained reports. One additional report was excluded during final eligibility reconciliation because adult data could not be separated from data for participants younger than 18 years. Thus, 25 reports were retained, representing 24 unique cohorts after accounting for the overlapping Jeung and Hong (2022) companion report. The three unretrieved reports remain separate from the eight full-text/final-eligibility exclusions.

The full-text exclusion log is provided in Appendix A. It lists the seven reports excluded at initial full-text assessment and the one additional report excluded during final eligibility reconciliation. The reasons are reproduced from the screening workbook: six reports involved mixed-age cohorts in which adult-only data could not be separated; one report involved a selected follicular-neoplasm cohort for which adult-only eligibility was not confirmed; and one further mixed-age report was excluded during final reconciliation.

Finally, 25 reports representing 24 unique cohorts were retained for the systematic review. One report (Jeung and Hong, 2022) was identified as a companion/overlapping report of the cohort reported by Słowińska-Klencka et al. (2020), and was not counted as an independent cohort. The entire report-selection process is represented in the PRISMA flow diagram (Figure 1).

**Figure 1:**
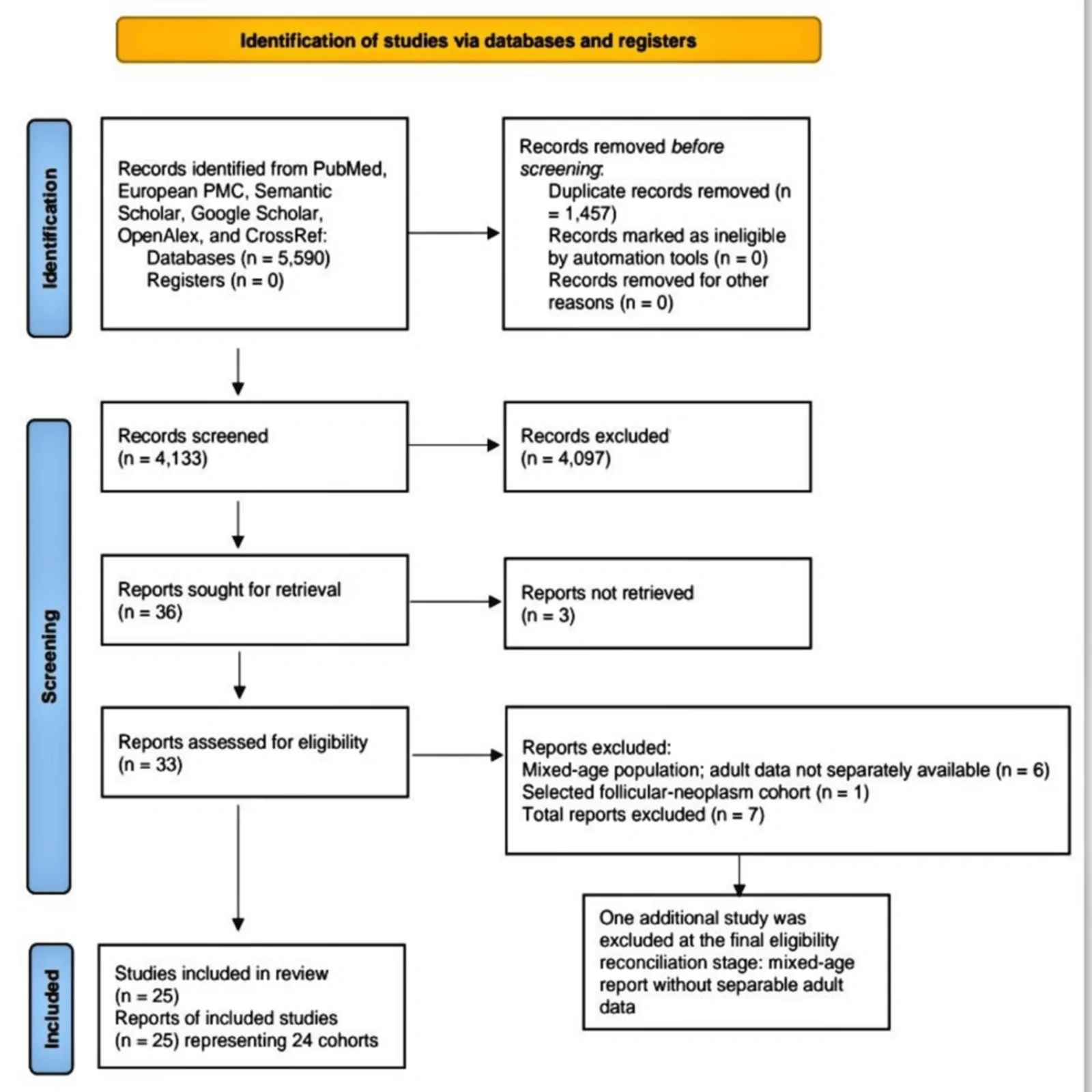
PRISMA 2020 Flow Diagram of the Systematic Review.

Data Synthesis and Statistical Analysis

A structured narrative synthesis was performed to compare the diagnostic performance of ACR TI-RADS and EU-TIRADS in adults with thyroid nodules. Where sufficient data were available, diagnostic accuracy measures, including sensitivity and specificity, were calculated from integer 2×2 contingency-table cells reported in the source articles or completed using complementary counts derived by subtraction from source-reported malignant and benign cohort totals. No 2×2 cells were estimated from rounded sensitivity or specificity values. For the Shen et al. category-5 comparison, FN and TN were derived from the reported totals of 773 malignant and 839 benign nodules after using the reported TP and FP counts for each system. These measures were presented at the individual-study level. A quantitative meta-analysis was not performed because of differences across studies in patient populations, diagnostic thresholds, reference standards, and reported outcomes, together with the limited availability of sufficiently comparable and verifiable data. Accordingly, no pooled estimates of sensitivity, specificity, DORs, or hierarchical summary receiver operating characteristic (HSROC) curves were generated. Findings were therefore synthesized descriptively, with attention to the diagnostic performance and clinical implications of each classification system.

Risk-of-Bias and Applicability Assessment

Risk of bias and applicability were assessed using the QUADAS-2 framework. Risk-of-bias domains were patient selection, index test, reference standard, and flow and timing. Applicability concerns were assessed for patient selection, index test, and reference standard. Each domain was judged as low, high, or unclear risk/concern, with supporting rationale recorded in the study-level assessment materials. The assessment was summarized for 24 unique cohorts, with seven domain-level judgments per cohort (168 judgments). The companion report by Jeung and Hong (2022), which overlaps with the cohort reported by Słowińska-Klencka et al. (2020), was not counted as a separate cohort in the QUADAS-2 summary. Ratings were checked against the original reports. QUADAS-2 was not converted into a numerical quality score.

Handling of Heterogeneity and Incomplete Reporting

The included studies differed in patient selection, cytology strata, diagnostic thresholds, reference standards, and verification pathways. Some reports evaluated selected surgical or biopsy cohorts or specific clinical subgroups, while others assessed FNA recommendations rather than a fixed TI-RADS category. The direction and magnitude of diagnostic performance may therefore depend on the study population, the threshold used to define a positive test, and the reference standard. Results were not treated as interchangeable across these settings. Where a study did not provide sufficient exact data for a compatible 2×2 table, its reported findings were retained for narrative description but were not used to derive unreported cell counts. No pooled estimate was produced across non-comparable thresholds or diagnostic constructs.

Certainty of Evidence

Certainty of evidence for the four primary matched category-5 diagnostic-accuracy outcomes was assessed using the Grading of Recommendations Assessment, Development, and Evaluation approach for DTA studies (GRADE-DTA), considering risk of bias, inconsistency, indirectness, imprecision, and publication bias where assessable. QUADAS-2 informed risk-of-bias judgments but was not converted into a numerical score. The two studies contributing verified exact 2×2 data were assessed separately for each system and outcome; no pooled estimate was generated. Confidence intervals were considered alongside the absence of prespecified clinical decision thresholds. Publication bias could not be meaningfully assessed with only two studies. The explicit outcome-level judgments and rationales are provided in Appendix B. FNA-recommendation analyses and other study-defined thresholds were not assigned a single cross-study certainty rating because their populations and diagnostic constructs were not sufficiently comparable.

Results

Study Selection

The six database searches yielded 5,590 records after within-database deduplication. Cross-database deduplication removed 1,457 duplicate records, leaving 4,133 records for title and abstract screening. Of these, 4,097 were excluded, and 36 reports were sought for retrieval. Three reports could not be retrieved, leaving 33 full-text reports assessed for eligibility. Seven reports were excluded at initial full-text screening, leaving 26 provisionally retained studies. One additional study was excluded because adult participants could not be separated from participants younger than 18 years. The final review set comprised 25 reports representing 24 unique cohorts; one companion report was not counted as an independent cohort. The search end date was October 12, 2026, and no publication-year restrictions were applied.

Characteristics of Included Studies and Outcome Availability

The 24-cohort set (represented by 25 reports) contains retrospective and prospective clinical diagnostic-performance studies, FNA-recommendation/rule-out studies, cytology-stratified cohorts, small-nodule and Hashimoto thyroiditis subgroups, and one computer-aided-diagnosis (CAD) analysis. Reported reference standards included histopathology, cytology/FNA, mixed cytology and histopathology, and clinical/imaging follow-up. Thresholds varied: some studies evaluated ACR category 5 versus EU-TIRADS category 5; others compared ACR TR4 or TR4-TR5 with EU-TIRADS category 5, category 3 or 4 thresholds, or size-dependent FNA recommendations. Single-system studies and non-equivalent endpoints were retained for descriptive purposes where eligible, but were not treated as direct evidence for the primary matched category-5 comparison. Therefore, the included reports do not constitute one homogeneous diagnostic-accuracy dataset.

Table 1 provides a traceable outcome-availability summary for each included study. Published sensitivity, specificity, predictive values, and AUCs are summarized in Table 2 when the source audit supports them. Exact confusion-matrix cells were not reconstructed from rounded metrics. Where the source itself has inconsistent denominators or reported accuracy, the discrepancy is retained as a limitation rather than silently corrected.

**Table 1:** Characteristics of Included Studies and Availability of Diagnostic Accuracy Outcomes. FNA: fine-needle aspiration; TIRADS: Thyroid Imaging Reporting and Data System; US: ultrasonography; AUC: area under the curve; ACR: American College of Radiology; CI: confidence interval; EU-TIRADS: European Thyroid Imaging and Reporting Data System; GEE: generalized estimating equations; PPV: positive predictive value; NPV: negative predictive value; FLUS: follicular lesion of undetermined significance; AUS: atypia of undetermined significance; SFN: suspicious for follicular neoplasm; SHT: suspicious for Hürthle cell tumor; TR: TI-RADS; TP: true positive; FP: false positive; FN: false negative; TN: true negative; ROC: receiver operating characteristic; CAD: computer-aided diagnosis; HT: Hashimoto thyroiditis

No.	Study	Endpoint/threshold	Exact 2×2 cells	Source-reported quantitative findings	Key limitation/interpretation
1	Seif et al., 2022 [11]	FNA recommendation/avoidable-biopsy endpoint	No/not verified	FNA-recommendation study; full matched category-5 2×2 data unavailable in the source audit.	Do not combine with fixed-category results; verify endpoint/denominator definitions before quantitative use.
2	Lu et al., 2023 [12]	Combined discriminant strategy using multiple TIRADS	No/not verified	Study examines a combined discriminant strategy using multiple TIRADS; no harmonized ACR-5/EU-5 comparison available in the extraction.	Combination/model endpoint differs from standalone systems; adult-only eligibility and quantitative endpoint were flagged in the audit.
3	Eidt et al., 2022/2023 [13]	Prospective FNA-US/FNA recommendation	No/not verified	Prospective FNA-US comparison; FNA recommendation/deferral is the principal comparable endpoint in current extraction.	Published summary contains a discrepancy in EU sensitivity; values were not pooled pending table reconciliation.
4	Sollmann et al., 2024 [14]	Fixed category thresholds and AUC; standalone and combined scintigraphy models	No/not verified	ACR category AUC 0.83 (95% CI 0.78-0.88); ACR score AUC 0.84 (0.79-0.89); EU-TIRADS AUC 0.78 (0.72-0.83). At ≥4, sensitivity/specificity: ACR 0.85/0.62 and EU 0.83/0.62. At category 5, ACR 0.48/0.97 and EU 0.67/0.81.	Selected histopathology cohort, 322 nodules/208 patients; GEE used for clustering. Exact 2×2 cells not available; do not reconstruct.
5	Borges et al., 2023 [15]	Surgical subset diagnostic accuracy; separate FNA recommendation outcomes	Yes - separate surgical subset	Surgical subset (n=75): ACR sensitivity 87.5%, specificity 27.1%, PPV 24.6%, NPV 88.9%; EU sensitivity 87.5%, specificity 18.6%, PPV 22.6%, NPV 84.6%.	Exact cells available for the selected surgical subset only; not harmonized to the primary category-5 comparison.
6	Jeung & Hong, 2022 [16] (companion report)	Cytology-stratified AUC and category results; companion-report results	No/not verified	Cytology-stratified AUCs: unequivocal cytology ACR 0.857/EU 0.855; FLUS/AUS 0.655/0.693; SFN/SHT 0.593/0.605; suspicious-for-malignancy 0.775/0.851. The companion report also reports overall AUCs of 0.771 (ACR) and 0.784 (EU).	The 2022 report appears to reuse the same 1,000-nodule/866-patient cohort and is treated as a companion report, not an independent cohort. No single overall 2×2 estimate is available.
7	Wang et al., 2026 [17]	Matched ACR TR5 vs EU-TIRADS 5; separate FNA recommendation analysis	Yes - matched primary	Matched category-5 exact cells: ACR 409/68/66/631 and EU 410/99/65/600 (TP/FP/FN/TN). Sensitivity 86.1% vs 86.3%; specificity 90.3% vs 85.8%; AUC 0.882 (0.862-0.900) vs 0.861 (0.840-0.880). FNA-decision sensitivity 45.7% vs 45.5%; specificity 76.4% vs 61.9%; FNA-decision AUC 0.610 vs 0.537.	Retrospective, selected biopsy/surgical verification. The published table reports EU-TIRADS specificity as 85.4% despite 600/699 corresponding to 85.8%; this review uses the integer counts and flags the source arithmetic discrepancy.
8	Shen et al., 2019 [18]	Matched ACR TR5 vs EU-TIRADS 5	Yes - matched primary	Matched category-5 exact cells: ACR 682/105/91/734 and EU 721/160/52/679 (TP/FP/FN/TN). Sensitivity 88.2% vs 93.3%; specificity 87.5% vs 80.9%. Reported AUC 0.879 (0.860-0.897) vs 0.872 (0.853-0.890); PPV 86.7% vs 81.9%; NPV 89.0% vs 92.9%.	Selected pathology-confirmed cohort; derived FN/TN are subtracted from reported cohort totals. Published text has small rounding discrepancies; the 2×2 table drives recalculated values.
9	Lee et al., 2021 [19]	Category ≥5/ROC in selected nondiagnostic-cytology surgical subgroup	No/not verified	Selected nondiagnostic/unsatisfactory-cytology surgical subgroup: AUC ACR 0.712 (0.606-0.803), EU 0.667 (0.559-0.764); pairwise AUC difference 0.045 (-0.012 to 0.102), p=0.1213.	Only 89 nodules surgically resected; selective verification/spectrum bias; no exact 2×2 cells extracted.
10	Tobcu et al., 2025 [20]	Study-reported diagnostic performance; separate FNA-indication endpoint	No/not verified	Table 3 reports ACR sensitivity 78.8%, specificity 58.4%, PPV 36.3%, NPV 90.2%, accuracy 63.1%; EU sensitivity 90.4%, specificity 48.6%, PPV 34.6%, NPV 94.4%, accuracy 58.2%.	ACR specificity differs slightly between the abstract and Table 3; table value retained and discrepancy flagged. Mixed reference standards; no exact matched category-5 cells.
11	Koc et al., 2020 [21]	Category ≥3, cytology-defined outcome	No/not verified	At category ≥3, ACR sensitivity 48.89%, specificity 60.63%, AUC 0.55 (0.51-0.60); EU sensitivity 86.67%, specificity 48.99%, AUC 0.73 (0.69-0.77).	Published accuracy values do not reconcile with sensitivity/specificity and denominator; cytology-defined outcome, not category 5.
12	Ha et al., 2021 [22]	FNA recommendation using size and guideline criteria	No/not verified	FNA indicated as positive: ACR sensitivity 76.1%, specificity 61.8%, PPV 32.5%, NPV 91.4%; EU sensitivity 84.6%, specificity 39.3%, PPV 25.2%, NPV 91.4%.	Large multicentre dataset; endpoint includes size-based biopsy rules, not fixed category threshold.
13	Mohan et al., 2024 [23]	Guideline-specific FNA decision criteria; prospective cohort	No/not verified	Prospective cohort of 625 nodules (89 malignant). ACR: sensitivity 93.26%, specificity 50.75%, PPV 23.92%, NPV 97.84%, accuracy 56.80%; EU: sensitivity 95.51%, specificity 26.68%, PPV 17.78%, NPV 97.28%, accuracy 36.48%. K-TIRADS results are not part of this two-system comparison.	Metrics are for guideline-specific FNA decisions and referral thresholds, not matched category-5 classification; mixed FNAC/histopathology reference.
14	Sharma et al., 2021 [24]	Diagnostic comparison across five guideline criteria	No/not verified	ACR-TIRADS: sensitivity 92.31% (75-99), specificity 40.23% (32-47), PPV 18.75%, NPV 97.22%, AUC 0.6627 (0.59-0.72). EU-TIRADS: sensitivity 76.92% (56-91), specificity 36.78% (29-44), PPV 15.38%, NPV 91.43%, AUC 0.5685 (0.47-0.65).	FNA-biopsy cohort of 200 patients; diagnostic analysis uses cytology and five different guideline criteria. This is not a matched category-5 comparison.
15	Schenke et al., 2020 [7]	Small nodules; ACR TR4-TR5 vs EU-TIRADS 5	No/not verified	Small nodules ≤10 mm: ACR TR4-TR5 sensitivity 100%, specificity 40.6%, PPV 65.0%, NPV 100%, accuracy 71.7%; EU-TIRADS 5 sensitivity 97.4%, specificity 49.3%, PPV 67.9%, NPV 94.4%, accuracy 74.5%.	Surgically selected cohort (76 malignant/69 benign) and unmatched thresholds; do not pool with the category-5-only comparison.
16	Seifert et al., 2021 [25]	ACR TR5 vs EU-TIRADS 5; published rounded metrics only	No/not verified	Reported ACR sensitivity 0.68 (0.61-0.74), specificity 0.80 (0.77-0.82), PPV 0.38, NPV 0.93, AUC 0.795 (0.759-0.831); EU sensitivity 0.84 (0.78-0.88), specificity 0.67 (0.64-0.70), PPV 0.32, NPV 0.96, AUC 0.800 (0.765-0.834).	Matched highest categories conceptually, but exact integer cross-tabs not located in source/supplement; narrative only, no reconstructed counts.
17	Słowińska-Klencka et al., 2020 [26] (companion report)	Cytology-stratified AUC and category results; companion-report results	No/not verified	Cytology-stratified AUCs: unequivocal cytology ACR 0.857/EU 0.855; FLUS/AUS 0.655/0.693; SFN/SHT 0.593/0.605; suspicious-for-malignancy 0.775/0.851. The companion report also reports overall AUCs of 0.771 (ACR) and 0.784 (EU).	The 2022 report appears to reuse the same 1,000-nodule/866-patient cohort and is treated as a companion report, not an independent cohort. No single overall 2×2 estimate is available.
18	Chambara et al., 2021 [27]	CAD-assisted category 4 performance	No/not verified	CAD default setting at category 4: ACR sensitivity 79.6%, specificity 43.0%, PPV 56.1%, NPV 69.7%, AUROC 0.72 (0.65-0.79); EU sensitivity 82.7%, specificity 42.1%, PPV 56.6%, NPV 72.6%, AUROC 0.65 (0.58-0.71).	CAD outputs; PPV/NPV/accuracy are not general-population estimates according to authors. Keep separate from routine-reader analysis.
19	Popova et al., 2021 [28]	Category performance; internal accuracy inconsistency	No/not verified	Abstract reports ACR sensitivity 97.2%, specificity 41.1%, PPV 19.0%, NPV 99.0%; EU sensitivity 97.2%, specificity 39.9%, PPV 18.7%, NPV 99.0%.	Internal inconsistency in reported accuracy (ACR 48.0%, EU 73.3%); do not use accuracy or reconstruct 2×2 cells.
20	Grani et al., 2019 [29]	FNA recommendation/biopsy deferral	No/not verified	FNA-deferral outcome: ACR sensitivity 83.3%, specificity 56.2%, PPV 12.8%, NPV 97.8%, AUC 0.70 (0.62-0.78), 53.4% biopsies avoided; EU sensitivity 86.1%, specificity 32.0%, PPV 8.9%, NPV 96.7%, AUC 0.59 (0.50-0.68), 30.7% biopsies avoided.	Biopsy-deferral rule combines category and size; no full exact 2×2 table; do not pool with category threshold results.
21	Grani et al., 2020 [30]	Age-stratified FNA recommendation/rule-out	No/not verified	Age ≤65: ACR sensitivity 83.67%, specificity 53.3%, PPV 13.6%, NPV 97.4%, AUROC 0.68; EU 85.7%, 26.4%, 9.3%, 95.4%, AUROC 0.56. Age >65: ACR 100%, 47.1%, 6.9%, 100%, AUROC 0.73; EU 100%, 25.5%, 5.0%, 100%, AUROC 0.63.	Age-stratified FNA rule-out outcomes; low malignancy prevalence in >65 subgroup and no complete exact 2×2 cells.
22	David et al., 2025 [31]	Observer-specific classifications; denominator inconsistency	No/not verified	Abstract: ACR sensitivity 100%, specificity 86%, PPV 43%, NPV 100%; EU sensitivity 100%, specificity 79%, PPV 33%, NPV 100%. High-experience observer table differs (ACR specificity 89%; EU 75.8%).	Source denominator mismatch and observer-specific estimates; retain both contexts as reported, not pooled.
23	Azzouz et al., 2021 [32]	ACR TR4/5 and EU categories 4/5; cytology subset	No/not verified	Cytology subset: ACR sensitivity 100% (95% CI 81-100), specificity 36.4% (23-51), PPV 37.8%, NPV 100%; EU sensitivity 100% (81-100), specificity 31.8% (20-46), PPV 36.1%, NPV 100%.	Small analytic subset after excluding Bethesda I/III/IV; summary denominators/PPV do not reconcile; no reconstructed cells.
24	Xu et al., 2019 [33]	ACR TR4 vs EU-TIRADS 5; unmatched thresholds	No/not verified	Unmatched thresholds: ACR TR4 positive AUC 0.846 (0.831-0.860), sensitivity 96.6%, specificity 52.9%, PPV 58.6%, NPV 95.8%; EU category 5 AUC 0.843 (0.828-0.859), sensitivity 83.2%, specificity 79.4%, PPV 73.5%, NPV 87.3%.	AUC comparison reported nonsignificant (p=0.601); TR4 vs category 5 is not a matched threshold.
25	Słowińska-Klencka et al., 2021 [34]	Hashimoto vs non-Hashimoto subgroups; shape-threshold sensitivity analysis	No/not verified	Hashimoto subgroup (AP>T): ACR sensitivity/specificity/AUC HT 49.4%/95.2%/0.791; non-HT 33.2%/95.2%/0.752. EU HT 71.3%/85.6%/0.817; non-HT 61.1%/85.5%/0.782.	Subgroup-specific; shape-threshold modifications materially change performance. Do not pool HT and non-HT or alternate thresholds.

**Table 2:** Matched Comparison of ACR TI-RADS Category 5 and EU-TIRADS Category 5: Study-Level Diagnostic Accuracy. TP: true positive; FP: false positive; FN: false negative; TN: true negative; CI: confidence interval; LR+: positive likelihood ratio; LR−: negative likelihood ratio; DOR: diagnostic odds ratio; ACR: American College of Radiology; TI-RADS: Thyroid Imaging Reporting and Data System; EU-TIRADS: European Thyroid Imaging and Reporting Data System

Study	System	TP/FP/FN/TN	Sensitivity (95% CI)	Specificity (95% CI)	LR+ (95% CI)	LR- (95% CI)	DOR (95% CI)
Wang et al., 2026 [17]	ACR TI-RADS	409/68/66/631	86.1% (82.7-88.9)	90.3% (87.8-92.3)	8.85 (7.04-11.13)	0.15 (0.12-0.19)	57.50 (40.09-82.49)
Wang et al., 2026 [17]	EU-TIRADS	410/99/65/600	86.3% (82.9-89.1)	85.8% (83.1-88.2)	6.09 (5.06-7.34)	0.16 (0.13-0.20)	38.23 (27.29-53.56)
Shen et al., 2019 [18]	ACR TI-RADS	682/105/91/734	88.2% (85.8-90.3)	87.5% (85.1-89.6)	7.05 (5.88-8.45)	0.13 (0.11-0.16)	52.39 (38.83-70.68)
Shen et al., 2019 [18]	EU-TIRADS	721/160/52/679	93.3% (91.3-94.8)	80.9% (78.1-83.4)	4.89 (4.25-5.63)	0.08 (0.06-0.11)	58.84 (42.30-81.84)

ACR TI-RADS Category 5 Versus EU-TIRADS Category 5

Although both systems were applied within the same study cohorts, the available reports provided separate marginal 2×2 tables rather than the joint cross-classification of each nodule by both systems. The numbers of nodules classified positive by ACR but negative by EU-TIRADS, and vice versa, were therefore unavailable. A paired comparison of sensitivity or specificity (for example, a McNemar-type analysis) could not be reconstructed from the marginal tables, and no between-system p-value was calculated by this review. Confidence-interval overlap is descriptive and was not used as a formal test of a difference. Any AUC comparison p-values cited in the Results are those reported by the original studies, not newly calculated paired tests.

The diagnostic unit of analysis in the primary category-5 comparison was the thyroid nodule, because the source studies reported TP, FP, FN, and TN counts by nodule classification. Patient counts were recorded separately whenever reported and were not treated as interchangeable with nodule denominators. Wang et al. (2026) [17] analyzed 1,174 nodules from 1,174 patients. Shen et al. (2019) [18] analyzed 1,612 nodules from 1,568 patients, indicating that some participants contributed more than one nodule. The available source information did not establish whether Shen et al. [18] adjusted the category-level diagnostic-accuracy estimates or their confidence intervals for within-patient clustering. Accordingly, its nodule-level estimates are reported as published/recalculated from nodule counts, with possible dependence between nodules acknowledged as a limitation. No patient-level cluster adjustment could be reconstructed from aggregate 2×2 data.

Two studies had verified exact 2×2 tables for the primary matched category-5 comparison: Wang et al. (2026) [17] and Shen et al. (2019) [18]. For each study and classification system, sensitivity was calculated as $\frac{\mathrm{TP}}{\mathrm{TP}+\mathrm{FN}}$, specificity as $\frac{\mathrm{TN}}{\mathrm{TN}+\mathrm{FP}}$, the positive likelihood ratio (LR+) as $\frac{\mathrm{Sensitivity}}{1-\mathrm{Specificity}}$, the negative likelihood ratio (LR-) as $\frac{1-\mathrm{Sensitivity}}{\mathrm{Specificity}}$, and the DOR as $\frac{\mathrm{TP}\times\mathrm{TN}}{\mathrm{FP}\times\mathrm{FN}}$. Wilson 95% confidence intervals were calculated for sensitivity and specificity; log-scale intervals were used for LR+, LR-, and DOR. Shen’s FN and TN cells were derived by subtracting the category-5 TP and FP counts from the reported totals of 773 malignant and 839 benign nodules; Wang’s cells were checked against the published category counts. The original articles contain small inconsistencies between some rounded published metrics and the integer counts; Table 2 consistently uses calculations from the verified integer cells. Estimates and confidence intervals are presented separately by study and system and are not pooled.

In Wang et al. (2026) [17], sensitivity was almost identical for ACR TI-RADS and EU-TIRADS (86.1% vs 86.3%), while specificity was higher for ACR TI-RADS (90.3% vs 85.8%). The reported AUCs were 0.882 (95% CI 0.862-0.900) for ACR TI-RADS and 0.861 (0.840-0.880) for EU-TIRADS; the source table reports a significant AUC difference (p=0.0031).

In Shen et al. (2019) [18], EU-TIRADS had higher sensitivity than ACR TI-RADS (93.3% vs 88.2%), whereas ACR TI-RADS had higher specificity (87.5% vs 80.9%). Reported AUCs were close (0.879 (0.860-0.897) vs 0.872 (0.853-0.890)); the study did not report a significant AUC difference between ACR and EU-TIRADS. A small discrepancy exists between some published rounded estimates and the exact-cell recalculation; the 2×2-based values are used consistently in Table 2.

Across the two studies, EU-TIRADS category 5 had a higher study-specific sensitivity, while ACR TI-RADS category 5 had a higher study-specific specificity. These are descriptive directions, not evidence of statistically significant between-system differences. The available marginal 2×2 tables do not provide the joint discordant classifications needed for a valid paired comparison; no between-system significance test was therefore performed by this review.

Other Fixed-Threshold, Subgroup, and AUC Findings

The studies below report useful diagnostic metrics but do not satisfy the same population/threshold/reference-standard conditions as the primary matched category-5 dataset. They are therefore reported separately rather than combined into a single pooled estimate (Table 3).

**Table 3:** Other Fixed-Threshold, Subgroup, and AUC Findings Across Included Studies. Predictive values are prevalence-dependent and are not directly comparable across cohorts with different malignancy prevalence, selection criteria, or reference standards. The AUC results also represent different estimands: standalone category classification, overall score discrimination, cytology-subgroup ROC curves, CAD output, and FNA-decision rules. These are not interchangeable and were not pooled. ACR: American College of Radiology; AUC: area under the curve; CI: confidence interval; EU-TIRADS: European Thyroid Imaging and Reporting Data System; GEE: generalized estimating equations; PPV: positive predictive value; NPV: negative predictive value; FNA: fine-needle aspiration; TI-RADS: Thyroid Imaging Reporting and Data System; CAD: computer-aided diagnosis; AUROC: area under the receiver operating characteristic curve; FLUS: follicular lesion of undetermined significance; AUS: atypia of undetermined significance; SFN: suspicious for follicular neoplasm; SHT: suspicious for Hürthle cell tumor; HT: Hashimoto thyroiditis

Study/year	Reported outcome metrics	Why separate/caveat
Sollmann et al., 2024 [14]	ACR category AUC 0.83 (95% CI 0.78-0.88); ACR score AUC 0.84 (0.79-0.89); EU-TIRADS AUC 0.78 (0.72-0.83). At ≥4, sensitivity/specificity: ACR 0.85/0.62 and EU 0.83/0.62. At category 5, ACR 0.48/0.97 and EU 0.67/0.81.	Selected histopathology cohort, 322 nodules/208 patients; GEE used for clustering. Exact 2×2 cells not available; do not reconstruct.
Borges et al., 2023 [15]	Surgical subset (n=75): ACR sensitivity 87.5%, specificity 27.1%, PPV 24.6%, NPV 88.9%; EU sensitivity 87.5%, specificity 18.6%, PPV 22.6%, NPV 84.6%.	Exact cells available for the selected surgical subset only; not harmonized to the primary category-5 comparison.
Lee et al., 2021 [19]	Selected nondiagnostic/unsatisfactory-cytology surgical subgroup: AUC ACR 0.712 (0.606-0.803), EU 0.667 (0.559-0.764); pairwise AUC difference 0.045 (-0.012 to 0.102), p=0.1213.	Only 89 nodules were surgically resected; selective verification/spectrum bias; no exact 2×2 cells extracted.
Tobcu et al., 2025 [20]	Table 3 reports ACR sensitivity 78.8%, specificity 58.4%, PPV 36.3%, NPV 90.2%, accuracy 63.1%; EU sensitivity 90.4%, specificity 48.6%, PPV 34.6%, NPV 94.4%, accuracy 58.2%.	ACR specificity differs slightly between the abstract and Table 3; table value retained and discrepancy flagged. Mixed reference standards; no exact matched category-5 cells.
Ha et al., 2021 [22]	FNA indicated as positive: ACR sensitivity 76.1%, specificity 61.8%, PPV 32.5%, NPV 91.4%; EU sensitivity 84.6%, specificity 39.3%, PPV 25.2%, NPV 91.4%.	Large multicentre dataset; endpoint includes size-based biopsy rules, not a fixed category threshold.
Sharma et al., 2021 [24]	ACR-TIRADS: sensitivity 92.31% (75-99), specificity 40.23% (32-47), PPV 18.75%, NPV 97.22%, AUC 0.6627 (0.59-0.72). EU-TIRADS: sensitivity 76.92% (56-91), specificity 36.78% (29-44), PPV 15.38%, NPV 91.43%, AUC 0.5685 (0.47-0.65).	FNA-biopsy cohort of 200 patients; diagnostic analysis uses cytology and five different guideline criteria. This is not a matched category-5 comparison.
Schenke et al., 2020 [7]	Small nodules ≤10 mm: ACR TR4-TR5 sensitivity 100%, specificity 40.6%, PPV 65.0%, NPV 100%, accuracy 71.7%; EU-TIRADS 5 sensitivity 97.4%, specificity 49.3%, PPV 67.9%, NPV 94.4%, accuracy 74.5%.	Surgically selected cohort (76 malignant/69 benign) and unmatched thresholds; do not pool with the category-5-only comparison.
Seifert et al., 2021 [25]	Reported ACR sensitivity 0.68 (0.61-0.74), specificity 0.80 (0.77-0.82), PPV 0.38, NPV 0.93, AUC 0.795 (0.759-0.831); EU sensitivity 0.84 (0.78-0.88), specificity 0.67 (0.64-0.70), PPV 0.32, NPV 0.96, AUC 0.800 (0.765-0.834).	Matched highest categories conceptually, but exact integer cross-tabs not located in source/supplement; narrative only, no reconstructed counts.
Słowińska-Klencka et al., 2020 [26]; companion report Jeung & Hong, 2022 [16]	Cytology-stratified AUCs: unequivocal cytology ACR 0.857/EU 0.855; FLUS/AUS 0.655/0.693; SFN/SHT 0.593/0.605; suspicious-for-malignancy 0.775/0.851. Companion report overall AUCs: ACR 0.771 and EU 0.784.	Same 1,000-nodule/866-patient cohort reported in both publications; counted once. Cytology-stratified/subgroup results are retained as companion-report information and are not independent evidence.
Chambara et al., 2021 [27]	CAD default setting at category 4: ACR sensitivity 79.6%, specificity 43.0%, PPV 56.1%, NPV 69.7%, AUROC 0.72 (0.65-0.79); EU sensitivity 82.7%, specificity 42.1%, PPV 56.6%, NPV 72.6%, AUROC 0.65 (0.58-0.71).	CAD outputs; PPV/NPV/accuracy are not general-population estimates according to the authors. Keep separate from routine-reader analysis.
Popova et al., 2021 [28]	Abstract reports ACR sensitivity 97.2%, specificity 41.1%, PPV 19.0%, NPV 99.0%; EU sensitivity 97.2%, specificity 39.9%, PPV 18.7%, NPV 99.0%.	Internal inconsistency in reported accuracy (ACR 48.0%, EU 73.3%); do not use accuracy or reconstruct 2×2 cells.
David et al., 2025 [31]	Abstract: ACR sensitivity 100%, specificity 86%, PPV 43%, NPV 100%; EU sensitivity 100%, specificity 79%, PPV 33%, NPV 100%. High-experience observer table differs (ACR specificity 89%; EU 75.8%).	Source denominator mismatch and observer-specific estimates; retain both contexts as reported, not pooled.
Azzouz et al., 2021 [32]	Cytology subset: ACR sensitivity 100% (95% CI 81-100), specificity 36.4% (23-51), PPV 37.8%, NPV 100%; EU sensitivity 100% (81-100), specificity 31.8% (20-46), PPV 36.1%, NPV 100%.	Small analytic subset after excluding Bethesda I/III/IV; summary denominators/PPV do not reconcile; no reconstructed cells.
Xu et al., 2019 [33]	Unmatched thresholds: ACR TR4 positive AUC 0.846 (0.831-0.860), sensitivity 96.6%, specificity 52.9%, PPV 58.6%, NPV 95.8%; EU category 5 AUC 0.843 (0.828-0.859), sensitivity 83.2%, specificity 79.4%, PPV 73.5%, NPV 87.3%.	AUC comparison reported nonsignificant (p=0.601); TR4 vs category 5 is not a matched threshold.
Słowińska-Klencka et al., 2021 [34]	Hashimoto subgroup (AP>T): ACR sensitivity/specificity/AUC HT 49.4%/95.2%/0.791; non-HT 33.2%/95.2%/0.752. EU HT 71.3%/85.6%/0.817; non-HT 61.1%/85.5%/0.782.	Subgroup-specific; shape-threshold modifications materially change performance. Do not pool HT and non-HT or alternate thresholds.

FNA Recommendation and Biopsy-Deferral Performance

FNA recommendation is a clinical decision endpoint that may incorporate category and nodule-size rules; it is not equivalent to testing whether a nodule is category 5. Results are therefore presented separately in Table 4.

**Table 4:** Diagnostic Performance of ACR TI-RADS and EU-TIRADS FNA Recommendation Criteria. ACR: American College of Radiology; TI-RADS: Thyroid Imaging Reporting and Data System; TP: true positive; FP: false positive; FN: false negative; TN: true negative; CI: confidence interval; LR+: positive likelihood ratio; LR-: negative likelihood ratio; DOR: diagnostic odds ratio; EU-TIRADS: European Thyroid Imaging and Reporting Data System; FNA: fine-needle aspiration

Study	System	TP/FP/FN/TN	Sensitivity (95% CI)	Specificity (95% CI)	LR+	LR-	DOR
Wang et al., 2026 [17]	ACR TI-RADS	217/165/258/534	45.7% (41.3-50.2)	76.4% (73.1-79.4)	1.94	0.71	2.72
Wang et al., 2026 [17]	EU-TIRADS	216/266/259/433	45.5% (41.0-50.0)	61.9% (58.3-65.5)	1.19	0.88	1.36
Ha et al., 2021 [22]	ACR TI-RADS	845/1756/266/2841	76.1% (73.5-78.5)	61.8% (60.4-63.2)	1.99	0.39	5.14
Ha et al., 2021 [22]	EU-TIRADS	940/2790/171/1807	84.6% (82.4-86.6)	39.3% (37.9-40.7)	1.39	0.39	3.56

For Wang et al. (2026) [17], FNA recommendation sensitivity was similar for ACR and EU-TIRADS (45.7% and 45.5%), while specificity was higher for ACR (76.4% vs 61.9%); FNA-decision AUCs were 0.610 and 0.537, respectively. For Ha et al. (2021) [22], EU-TIRADS FNA criteria had higher sensitivity (84.6% vs 76.1%) but lower specificity (39.3% vs 61.8%) than ACR TI-RADS. These study-level findings were not pooled because the cohorts, reference standards, and size-based biopsy rules differ.

Grani et al. (2019) [29] reported that ACR criteria would avoid 53.4% of biopsies compared with 30.7% under EU-TIRADS; corresponding sensitivity/specificity were 83.3%/56.2% and 86.1%/32.0%. Grani et al. (2020) [30] reported age-stratified rule-out results; ACR specificity and area under the receiver operating characteristic curve (AUROC) were higher in both age strata. These studies remain separate because the decision rule includes nodule size and/or age stratification.

Risk of Bias and Applicability

The final QUADAS-2 assessment covers 24 unique cohorts, with seven domain-level judgments per cohort (168 judgments). The overlapping Jeung and Hong (2022) [16] companion report was not counted as an independent cohort; the primary cohort report by Słowińska-Klencka et al. (2020) [26] is represented in Table 5. Ratings were checked against the original reports, and “Unclear” was retained where the published information did not resolve the relevant signalling questions.

**Table 5:** QUADAS-2 Risk-of-Bias and Applicability Assessments for Included Studies. RoB: risk of bias; QUADAS-2: Quality Assessment of Diagnostic Accuracy Studies 2

No.	Study	Patient selection RoB	Index test RoB	Reference standard RoB	Flow/timing RoB	Patient applicability	Index-test applicability	Reference-standard applicability
1	Seif et al., 2022 [11]	High	Unclear	Unclear	High	Low	Unclear	Unclear
2	Lu et al., 2023 [12]	Unclear	Unclear	Unclear	Unclear	Unclear	Unclear	Unclear
3	Eidt et al., 2022/2023 [13]	Low	Unclear	Unclear	High	Low	Low	Unclear
4	Sollmann et al., 2024 [14]	Unclear	Unclear	Low	Unclear	Unclear	Low	Low
5	Borges et al., 2023 [15]	High	Unclear	Low	High	High	Unclear	Low
6	Wang et al., 2026 [17]	Unclear	Unclear	Low	Unclear	Unclear	Low	Low
7	Shen et al., 2019 [18]	High	Unclear	Unclear	High	Low	Unclear	Unclear
8	Lee et al., 2021 [19]	High	Unclear	Low	Unclear	High	Low	Low
9	Tobcu et al., 2025 [20]	High	Unclear	Low	High	High	Unclear	Low
10	Koc et al., 2020 [21]	Unclear	Unclear	High	High	Unclear	Unclear	Low
11	Ha et al., 2021 [22]	Unclear	Unclear	Unclear	Unclear	Low	Unclear	Unclear
12	Mohan et al., 2024 [23]	High	Unclear	Unclear	High	Low	Unclear	Unclear
13	Sharma et al., 2021 [24]	Unclear	High	Unclear	Unclear	Unclear	High	Unclear
14	Schenke et al., 2020 [7]	Unclear	Unclear	Unclear	Unclear	High	Unclear	Unclear
15	Seifert et al., 2021 [25]	Unclear	Unclear	Unclear	Unclear	Unclear	Unclear	Unclear
16	Słowińska-Klencka et al., 2020 [26]	Unclear	Unclear	Unclear	Unclear	Unclear	Unclear	Unclear
17	Chambara et al., 2021 [27]	High	Unclear	Low	Unclear	High	High	Low
18	Popova et al., 2021 [28]	Unclear	High	Unclear	Unclear	Low	High	Unclear
19	Grani et al., 2019 [29]	Unclear	Unclear	High	High	Unclear	Low	Unclear
20	Grani et al., 2020 [30]	Unclear	Unclear	Unclear	Unclear	High	Low	Unclear
21	David et al., 2025 [31]	Unclear	High	Unclear	High	Unclear	High	Unclear
22	Azzouz et al., 2021 [32]	High	Unclear	Unclear	High	High	Unclear	Unclear
23	Xu et al., 2019 [33]	Unclear	High	Unclear	Unclear	Unclear	High	Unclear
24	Słowińska-Klencka et al., 2021 [34]	Unclear	High	Unclear	Unclear	High	High	Unclear

Among the 168 domain judgments for the 24 unique cohorts, 103 were “Unclear,” 39 were “High,” and 26 were “Low.” “Unclear” is retained as a substantive judgment where reporting is insufficient, not as an unfinished assessment. Recurring concerns included selected surgical/biopsy/cytology populations, partial or differential verification, unclear blinding, mixed reference standards, unmatched thresholds, CAD-assisted index tests, and possible clustering of multiple nodules within patients. These issues limit direct comparison and generalizability.

Table 5 presents the final study-level QUADAS-2 judgments for the 24 unique cohorts. Figure 2 provides the traffic-light plot, and Figure 3 summarizes the distribution of judgments across the seven QUADAS-2 domains. Nodule and patient denominators, and whether adjustment for clustering was identifiable from the available source information, are summarized in Appendix C.

**Figure 2:**
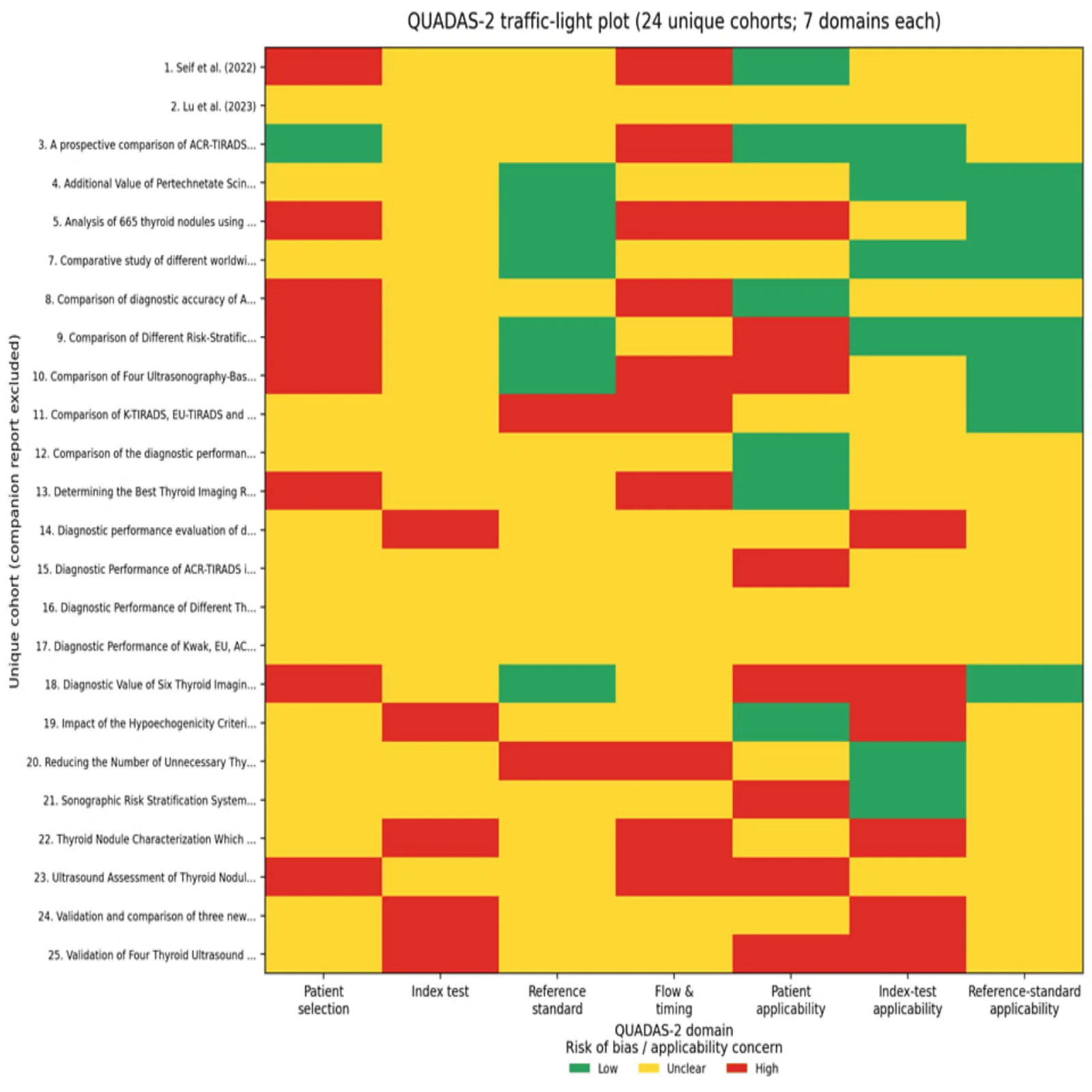
QUADAS-2 Traffic-Light Plot for 24 Unique Cohorts (168 Domain Judgments). The studies included are Seif et al. [11]; Lu et al. [12]; Eidt et al. [13]; Sollmann et al. [14]; Borges et al. [15]; Wang et al. [17]; Shen et al. [18]; Lee et al. [19]; Tobcu et al. [20]; Koc et al. [21]; Ha et al. [22]; Mohan et al. [23]; Sharma et al. [24]; Schenke et al. [7]; Seifert et al. [25]; Słowińska-Klencka et al. [26]; Chambara et al. [27]; Popova et al. [28]; Grani et al. [29]; Grani et al. [30]; David et al. [31]; Azzouz et al. [32]; Xu et al. [33]; and Słowińska-Klencka et al. [34]. The overlapping Jeung and Hong (2022) [16] companion report was not counted as a separate cohort. QUADAS-2: Quality Assessment of Diagnostic Accuracy Studies 2

**Figure 3:**
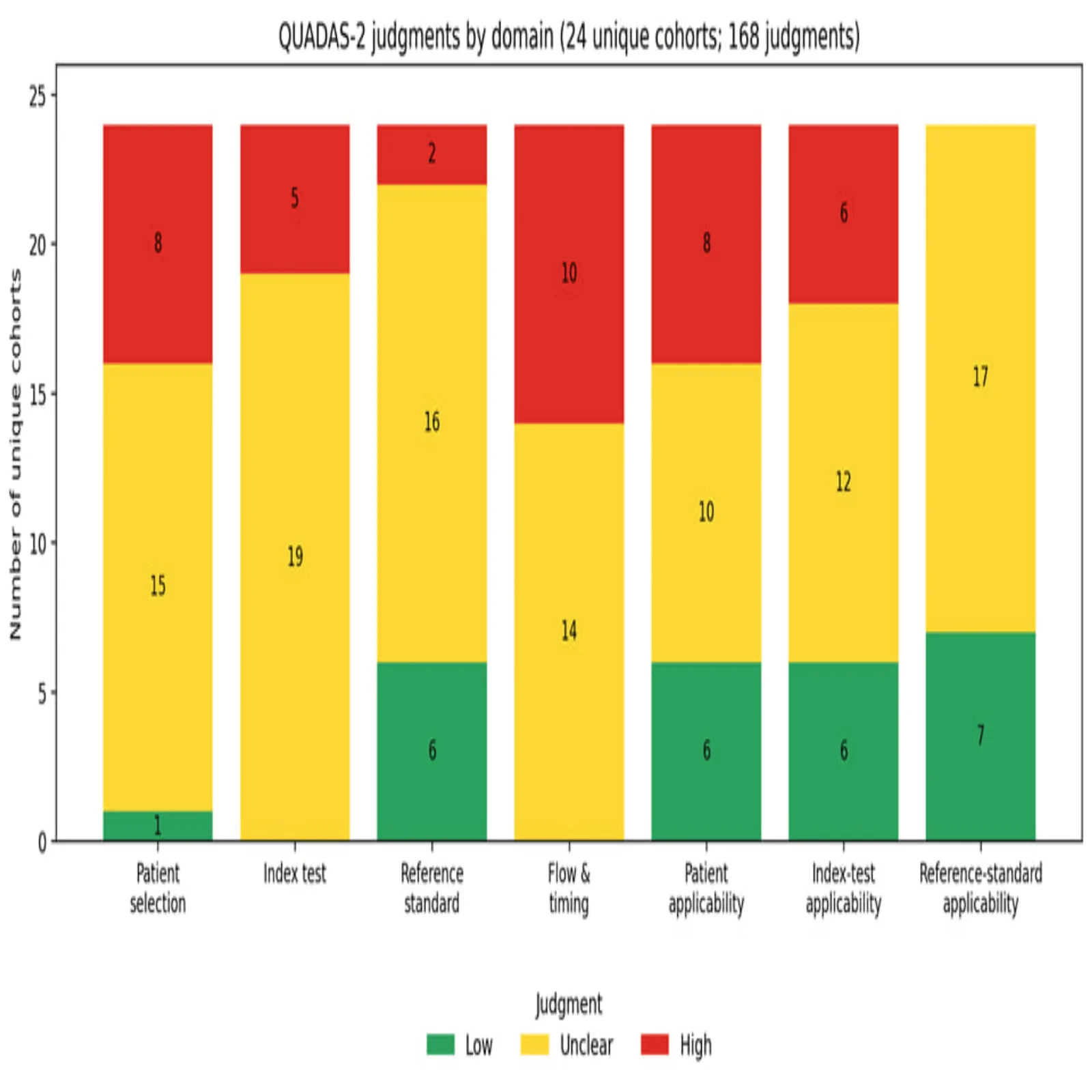
Distribution of Low, Unclear, and High QUADAS-2 Judgments Across the Seven Domains for 24 Unique Cohorts. QUADAS-2: Quality Assessment of Diagnostic Accuracy Studies 2

Certainty of Evidence

GRADE-DTA certainty was rated moderate for ACR category-5 sensitivity, ACR category-5 specificity, and EU-TIRADS category-5 specificity, and low for EU-TIRADS category-5 sensitivity. All four ratings were downgraded for serious risk-of-bias concerns in the two contributing studies; EU-TIRADS sensitivity was downgraded a further level for inconsistency between the study estimates. These ratings are qualitative and apply to the study-specific evidence base, not to pooled estimates. Publication bias could not be assessed reliably with only two studies. The outcome-level judgments and supporting rationales are presented in Appendix B. FNA-recommendation outcomes and other study-defined thresholds were not assigned a combined certainty rating because the endpoints and populations were not sufficiently comparable.

Quantitative Synthesis Decision and Summary

No pooled sensitivity, specificity, DOR, or HSROC estimate was calculated. Only two studies supplied verified paired exact 2×2 data for the prespecified matched category-5 comparison. The remaining studies reported different thresholds, selected populations, subgroup-specific estimates, FNA decisions, CAD results, rounded metrics without exact cells, or internally inconsistent denominators/accuracy values. A bivariate/HSROC model based on two studies would not provide a stable estimate of between-study variability. No missing TP/FP/FN/TN cells were inferred from rounded percentages.

The evidence supports a cautious descriptive conclusion: in the two verified matched category-5 studies, EU-TIRADS had higher sensitivity and ACR TI-RADS had higher specificity. Other studies show similar sensitivity-specificity trade-offs in some settings, but their differing thresholds, populations, reference standards, and endpoints prevent a single global claim of superiority. AUC and predictive values were reported study by study where available, not pooled across incompatible estimands.

Discussion

This systematic review analyzed the diagnostic performance of the ACR TI-RADS vs. the EU-TIRADS classification scheme developed by the European Thyroid Association for the detection of thyroid cancer in patients with thyroid nodules. Twenty-five articles have been included in the systematic review. The primary comparative analysis focused on the matched category-5 threshold, for which exact, verified 2 × 2 diagnostic data were available from two studies. The results indicate that the diagnostic performance of both TI-RADS systems is dependent on the threshold chosen to define a positive test result and the target population of interest. While both diagnostic tools have shown considerable diagnostic performance, differences between their sensitivities and specificities have been observed, and there was no basis for calculating a pooled estimate. This review demonstrates the necessity of distinguishing between the diagnostic performance of a category of classification and its clinical value.

Principal Findings and Comparison of the Two Classification Systems

In the primary matched category-5 comparison, Wang et al. reported similar sensitivities for ACR TI-RADS and EU-TIRADS (86.1% and 86.3%, respectively), with higher specificity for ACR TI-RADS (90.3% vs 85.8%). Their reported AUCs were 0.882 and 0.861, respectively, with a statistically significant difference reported by the original investigators. Shen et al. reported higher sensitivity for EU-TIRADS than ACR TI-RADS (93.3% vs 88.2%), whereas specificity was higher for ACR TI-RADS (87.5% vs 80.9%); the AUCs were close (0.879 and 0.872), without a significant difference in the original report. Thus, both studies showed the same descriptive direction for the highest-risk categories - greater sensitivity for EU-TIRADS 5 and greater specificity for ACR TR5 - but the size of the differences varied. These are study-specific marginal estimates, not a pooled or paired comparison: the available 2×2 tables do not identify the nodules classified discordantly by the two systems.

The direction of the findings is broadly consistent with earlier category-based meta-analyses, although those reviews provide indirect context rather than a direct test of the two systems in the same nodules. A meta-analysis by Kim et al. (2020), focused on category 4 or 5 thresholds across 34 studies and 37,585 nodules, found a trend toward higher sensitivity for EU-TIRADS at category 5 (78% vs 70% for ACR TI-RADS), while specificity estimates were similar (89% for both); differences between systems were not statistically significant [35]. These pooled estimates are compatible with the direction observed in the two matched studies in the present review, but they combine different study sets, populations, reference standards, and threshold implementations. They should not be interpreted as paired comparisons or as independent confirmation of superiority.

Interpretation of the Sensitivity-Specificity Trade-Off

Taken together, the previous reviews answer different questions: category-based diagnostic meta-analyses estimate average accuracy across heterogeneous study sets; biopsy-criteria reviews evaluate management decisions that incorporate nodule size; and category-distribution reviews compare how systems allocate nodules and assign malignancy risk. None of these designs substitutes for a paired head-to-head analysis of the same adult nodules at explicitly defined category thresholds. The present review adds a focused description of the two studies with verified exact 2×2 data for ACR TR5 versus EU-TIRADS 5, while remaining unable to establish statistical superiority because joint discordant classifications were not available.

The sensitivity-specificity trade-off is clinically relevant. Higher sensitivity may reduce missed malignancies but can increase false-positive classifications, additional imaging, biopsies, and detection of indolent lesions. Higher specificity may reduce unnecessary investigations but can increase false-negative results. Greater sensitivity is therefore not invariably beneficial: detection of small, indolent papillary thyroid microcarcinomas may contribute to overdiagnosis and overtreatment if ultrasound findings are interpreted without considering clinical context, nodule size, patient preferences, and the natural history of the disease.

Li et al. evaluated ACR TI-RADS alone in 16 studies involving 18,614 patients and 21,882 nodules, reporting pooled sensitivity of 89% and specificity of 70% across the diagnostic thresholds included in that review [36]. This provides a broad benchmark for ACR TI-RADS but does not directly compare ACR TI-RADS with EU-TIRADS, and its pooled estimates should not be equated with the category-5-specific results in the present review.

Comparison With Larger Head-to-Head Evaluations

Piticchio et al. (2024) conducted a head-to-head systematic review of seven studies comprising 41,332 nodules assessed with ACR-, EU-, and K-TIRADS [37]. Importantly, its principal outcomes were the distribution of nodules across risk categories and the malignancy risk assigned to those categories, rather than pooled sensitivity and specificity at a common category-5 diagnostic threshold. The review found differences in the distribution of low-risk nodules and in category-specific malignancy-risk estimates, while the distributions of intermediate- and high-risk categories were more similar. This work supports the clinical relevance of category calibration and classification differences, but it does not establish which system is more accurate at the matched highest-risk threshold evaluated in the present review.

Jin et al. (2023) retrospectively evaluated 3,438 nodules in 3,013 patients using C-TIRADS, ACR TI-RADS, and EU-TIRADS [38]. Across the study's selected diagnostic and management definitions, ACR TI-RADS had higher sensitivity than EU-TIRADS (89.1% vs 78.4%), whereas EU-TIRADS had higher specificity (78.9% vs 69.5%); the AUCs were 0.844 and 0.802, respectively. This direction differs from the two primary matched category-5 studies in the present review. The difference should not be interpreted as a contradiction: Jin et al. evaluated broader classification and FNA-management performance in a single retrospective cohort, whereas the primary analysis here isolates the respective highest-risk categories and requires verified integer 2×2 data. The positivity thresholds and clinical endpoints are therefore not identical. This example reinforces that the relative performance of the systems depends on the threshold and outcome being evaluated.

Threshold Effect and Non-equivalence of the Highest-Risk Categories

The apparent differences between ACR TR5 and EU-TIRADS 5 should be interpreted in light of how each category is defined. ACR TR5 is reached when the summed score across composition, echogenicity, shape, margins, and echogenic foci is at least 7 points. EU-TIRADS 5, in contrast, is assigned when at least one high-suspicion feature is present, such as a non-oval shape, irregular margins, microcalcifications, or marked hypoechogenicity. These rules are not interchangeable: a nodule may meet the EU-TIRADS 5 pattern definition without accumulating 7 ACR points, or may reach ACR TR5 through a combination of features that does not map one-to-one to the EU-TIRADS 5 definition. Consequently, the term “matched category 5” denotes the comparison of the highest-risk category in each system, not a biologically or feature-level equivalent threshold. The observed sensitivity-specificity trade-off may therefore reflect, at least in part, the different operating thresholds and feature combinations used by the systems, in addition to differences in study populations and reference standards. The two available studies do not provide patient/nodule-level cross-classification data sufficient to isolate the contribution of the threshold definitions or quantify a threshold effect independently.

Diagnostic Categories Versus FNA-Recommendation Outcomes

One of the key observations made in the current literature review is the necessity to differentiate between the performance of diagnostic categories and the performance of FNA recommendation rules. In the case of a category-based threshold, the nodule is classified depending on its ultrasound characteristics, while in the case of an FNA recommendation, there are other parameters taken into account, such as nodule size and criteria for management according to the guidelines.

Papers used in the current literature review exemplify this concept well. For instance, Wang et al. documented FNA decision sensitivities of 45.7% for ACR TI-RADS and 45.5% for EU-TIRADS, along with specificities of 76.4% and 61.9%, respectively. Such results vary greatly from the estimates of sensitivities calculated using the matched category-5 cutoff. Other papers included in the review have reported similar findings of high sensitivity and relatively low specificity for guidelines-based FNA decisions. It would be inaccurate to consider such findings as an indication of suboptimal performance of the system; on the contrary, the differences are due to the dissimilarity in the purpose of size-based and category-based management criteria and fixed high-risk categories, respectively.

Evidence comparing ACR TI-RADS with other management guidelines also illustrates why diagnostic classification and biopsy recommendations should not be conflated. Zhang et al. (2020) systematically compared ACR TI-RADS with the American Thyroid Association guidelines, reporting pooled sensitivity of 84% and specificity of 67% for ACR TI-RADS, compared with 89% and 46% for ATA [39]. Although this review did not evaluate EU-TIRADS, it demonstrates how guideline structure and biopsy thresholds can shift the sensitivity-specificity balance. It is therefore supportive background evidence, not a direct comparator for the ACR-versus-EU category-5 question.

Another meta-analysis by Kim et al. (2020), synthesizing 29 studies comprising 33,748 nodules, reported category-5 pooled sensitivity/specificity of 66%/91% for ACR TI-RADS and 82%/90% for EU-TIRADS; the authors considered overall performance across the systems comparable despite substantial between-study heterogeneity [40].

Our findings should also be interpreted in the context of previous systematic reviews and meta-analyses. Another meta-analysis found no statistically significant difference among ACR, EU, and Korean TI-RADS at category 5, despite a trend toward higher sensitivity for EU-TIRADS [35]. Li et al. reported an ACR-only synthesis, while Piticchio et al. examined category distribution and risk calibration rather than providing a directly comparable pooled category-5 sensitivity/specificity estimate [36,37]. Kim et al. included 29 studies and 33,748 nodules; at category 5, pooled sensitivity and specificity were 66% and 91% for ACR TI-RADS and 82% and 90% for EU-TIRADS, respectively [40]. These reviews provide broader pooled or network comparisons, but their study sets, system-specific optimal thresholds, and aggregate populations do not substitute for a same-cohort comparison using the respective highest-risk categories. Differences in case mix, malignancy prevalence, reference standards, and threshold definitions limit direct comparison between pooled estimates and the individual head-to-head studies in the present review. A summary of the relevant previously published systematic reviews and meta-analyses, including their evidence bases, principal findings, and relationship to the present review, is provided in Appendix H.

Sources of Heterogeneity and Methodological Considerations

Several clinical and methodological factors may explain the variation in diagnostic performance across the included studies. Patient selection differed: some studies enrolled unselected ultrasound populations, whereas others included patients referred for FNA, surgery, or specific cytopathology categories. Surgical and biopsy cohorts may have higher malignancy prevalence and a different disease spectrum than routine clinical populations, limiting generalizability.

Furthermore, different references were used in the verification process. Histopathology could be available to verify nodules removed surgically, while non-surgical nodules could be verified using cytological results or follow-up. When verification was based on the initial classification of ultrasound or suspicion of disease, differential verification and selection biases could affect the estimation of sensitivity and specificity. This current review, thus, maintained individual study limitations and did not consider all reported estimates equally.

Third, variations in how the positive result is defined have been a considerable source of heterogeneity in methodology. While some studies employed the highest risk category, others included more than one category, and still others assessed the size-dependent FNA recommendations. Modifications in the scoring criteria, computer-assisted evaluation, observer-specific classification, and subgroup analysis have created additional heterogeneity in the methodology, thus making a quantitative comparison impossible. For the purpose of the main analysis in the current review, only the matched category 5 comparison with available 2×2 data was selected.

Moreover, some discrepancies between the published values with rounded data and actual integer numbers were found, as well as some inconsistencies within the selected papers. If there was enough evidence to calculate exact contingency table cells, then such cells were used for calculations instead of constructing integer numbers out of the rounded values of sensitivity and specificity. This is the safer approach, although it leads to the situation where some potentially useful studies have been left only for descriptive analysis. This approach clarifies why a pooled value was not calculated.

Clinical Implications

The conclusions drawn have certain implications in clinical practice. In the first place, the selection of ACR TI-RADS vs. EU-TIRADS should take into account the aim of clinical application, the threshold being used, the features of the specific patient population, and biopsy-related specifics of each particular locality. The system that has more sensitivity will be preferred if prevention of undiagnosed malignancy becomes the main goal, while higher specificity will be important if reducing the number of invasive diagnostic procedures becomes an aim. This will have to be done, taking into consideration possible effects of false-positive and false-negative findings.

Clinicians should not assume that different TI-RADS systems will interpret a category in the same way. Different categories use varying combinations of features in their definitions and are associated with different risk levels. The same nodule might belong to different categories, depending on the classification system. The data provided by Piticchio et al. once again prove this statement [37]. Therefore, standardized application of a chosen system, including the reporting of the classification and the threshold used, will be necessary for communication between radiologists and endocrinologists.

Third, it would be premature to conclude based on the current findings that one system is better at minimizing unnecessary biopsies or improving patient outcomes compared with the other system. To do so, more studies are needed to show that the differences between the two systems are associated with different management practices, biopsy rates, missed malignancies, subsequent tests, and patient outcomes.

Strengths and Limitations

One advantage of this review is the wide search in six sources and the selection of studies assessing ACR TI-RADS versus EU-TIRADS for adults. In this review, diagnostic category thresholds as well as FNA decisions according to guidelines have been taken into account while keeping them separate. Data at the study level have been verified based on source reports, and a risk-of-bias assessment has been performed in accordance with QUADAS-2 across all 24 unique cohorts. The main analysis was conducted only based on studies with verifiable integer contingency data.

Several limitations should be considered. First, only two studies provided verified integer 2×2 data for the primary matched category-5 comparison; therefore, pooled diagnostic properties could not be estimated, and statistical superiority could not be established. Although the systems were evaluated in the same cohorts, paired discordant classifications were not reported, preventing a valid paired comparison from the available marginal tables. Second, ACR TR5 and EU-TIRADS 5 are the respective highest-risk categories but are not feature-level equivalent thresholds: ACR TR5 requires ≥7 cumulative points, whereas EU-TIRADS 5 requires at least one high-suspicion feature. The observed differences may partly reflect this threshold effect. Third, the primary diagnostic data were nodule-level rather than patient-level. Wang et al. included 1,174 nodules from 1,174 patients, whereas Shen et al. included 1,612 nodules from 1,568 patients. For Shen et al., the available source information did not establish whether within-patient clustering was accounted for in the category-specific estimates or confidence intervals. Aggregate 2×2 data do not permit a retrospective cluster correction; therefore, precision may be overstated if multiple nodules from the same patient were treated as independent. Fourth, the included studies varied in patient selection, malignancy prevalence, reference standards, thresholds, and endpoints. Fifth, reference lists of included reports were manually screened and checked against the deduplicated library; however, Embase, Scopus, Web of Science, and the Cochrane Library could not be searched because access was unavailable, which may have resulted in missed eligible studies. Sixth, the review was not registered in PROSPERO, and no prospectively registered protocol was available. Seventh, all three authors participated in title/abstract screening, full-text assessment, and data extraction, with disagreements resolved through discussion; the project records do not establish that each stage was conducted independently in duplicate. Eighth, Jeung and Hong (2022) was treated as a companion/overlapping report of the cohort reported by Słowińska-Klencka et al. (2020) and was not counted as an independent cohort. Finally, selection and verification bias cannot be excluded in selected surgical/biopsy cohorts, and several reports provided rounded metrics or incomplete denominators. Tan et al. (2020), identified through reference-list screening, was not included because the accessible report did not establish an adult-only study population; selective FNA verification also limited its suitability for the primary diagnostic-accuracy synthesis.

The additional studies had different objectives and methods, including comparisons of category distributions, FNA recommendations, other guideline systems, and CAD. Their findings are useful context but should not be interpreted as directly comparable estimates of the matched category-5 diagnostic accuracy evaluated in the primary analysis.

Recommendations for Future Research

Future research on these two TI-RADS classifications should compare their performance in the same group of adult consecutive patients, using pre-defined and clinically meaningful positivity levels. Such studies should provide the complete 2 × 2 tables with confidence intervals, prevalence of malignancy, nodule sizes, reference standard information, and percentage of patients who were evaluated histologically, cytologically, or by follow-up. In cases where both classifications are used for the same nodules, the paired analysis should be provided to allow proper statistical comparison of results, taking into account the correlation.

Furthermore, the future research should compare the diagnostic performance of the classification and recommendation for the FNA based on nodule size, reporting the results for both of them whenever it is possible. The analysis of the number of unnecessary biopsies and cases of missed malignancy would provide a much more clinically meaningful comparison than the comparison of just sensitivity and specificity. Finally, the well-powered multicenter study with standardized criteria would answer the question about the consistency of the results in different countries.

Overall, both ACR TI-RADS and EU-TIRADS are useful ultrasound-based risk-stratification systems. In the two studies with verified data at the highest-risk-category comparison (ACR TR5 versus EU-TIRADS 5), EU-TIRADS had higher sensitivity and ACR TI-RADS had higher specificity. The sensitivity difference was negligible in Wang et al. (86.3% vs 86.1%) and larger in Shen et al. (93.3% vs 88.2%). Because only two studies were available, the comparisons were descriptive, paired discordant classifications were unavailable, and the wider evidence was heterogeneous; neither system can be considered superior on the basis of this review.

## Conclusions

In this systematic review, both ACR TI-RADS and EU-TIRADS demonstrated clinically relevant diagnostic performance for thyroid nodule malignancy risk stratification. In the two studies with verified data at the highest-risk-category comparison (ACR TR5 versus EU-TIRADS 5), EU-TIRADS showed higher sensitivity and ACR TI-RADS showed higher specificity, although the sensitivity difference was minimal in one study. The limited number of direct comparisons, absence of paired discordant data, and heterogeneity across the broader evidence prevent a conclusion that either system is superior.

## Appendices

Appendix A 

**Table 6:** Full-Text Exclusion and Retrieval Log. ACR: American College of Radiology; TI-RADS: Thyroid Imaging Reporting and Data System; EU-TIRADS: European Thyroid Imaging and Reporting Data System; KSThR: Korean Society of Thyroid Radiology

No.	Report	Recorded reason for exclusion
1	Assessment of perinodular stiffness in differentiating malignant from benign thyroid nodules	Mixed-age cohort (age range 16-77 years); adult-only data could not be separated.
2	Diagnostic performance rates of the ACR-TIRADS and EU-TIRADS based on histopathological evidence	Mixed-age cohort (age range 17-78 years); adult-only data could not be separated.
3	Differences among the Thyroid Imaging Reporting and Data System proposed by Korean, the American College of Radiology and the European Thyroid Association in the diagnostic performance of thyroid nodules	Mixed-age cohort (age range 14-88 years); adult-only data could not be separated.
4	Effect of the categorization method on the diagnostic performance of ultrasound risk stratification systems for thyroid nodules	Mixed-age cohort (age range 7-82 years); adult-only data could not be separated.
5	Interrater Reliability of Various Thyroid Imaging Reporting and Data System (TIRADS) Classifications for Differentiating Benign from Malignant Thyroid Nodules	Mixed-age cohort (age range 10-75 years); adult-only data could not be separated.
6	Performance of current ultrasound-based malignancy risk stratification systems for thyroid nodules in patients with follicular neoplasms	Mixed-age cohort (age range 3-82 years); adult-only data could not be separated; selected follicular-neoplasm population.
7	Diagnostic performance of six ultrasound-based risk stratification systems in thyroid follicular neoplasm: A retrospective multi-center study (Yang et al., 2022)	Adult-only eligibility was not confirmed; the study examined a selected follicular adenoma-versus-carcinoma cohort rather than a general thyroid-nodule population. Exclusion was confirmed by the review team.
8	Explore the Diagnostic Efficiency of Chinese Thyroid Imaging Reporting and Data Systems by Comparing With the Other Four Systems (ACR TI-RADS, Kwak-TIRADS, KSThR-TIRADS, and EU-TIRADS): A Single-Center Study	Mixed-age cohort (age range 10-78 years); adult-only data could not be separated. This was excluded during final eligibility reconciliation.

Appendix B 

**Table 7:** GRADE-DTA Evidence Profile for the Matched Category-5 Comparison. ACR: American College of Radiology; TI-RADS: Thyroid Imaging Reporting and Data System; EU-TIRADS: European Thyroid Imaging and Reporting Data System; GRADE-DTA: Grading of Recommendations Assessment, Development, and Evaluation approach for diagnostic test accuracy studies; CI: confidence interval

Outcome	Evidence	Risk of bias	Inconsistency	Indirectness	Imprecision	Publication bias	Certainty	Rationale
ACR TI-RADS category 5 - sensitivity	Wang 2026; Shen 2019 (2 studies)	Serious concern; downgrade 1 level. Shen had high risk of bias in patient selection; Wang had unclear risk in patient selection and index test.	No downgrade: estimates were close (86.1% and 88.2%); only two studies, so heterogeneity cannot be estimated reliably.	No downgrade for the narrowly defined category-5 construct; study settings and selection limit generalizability.	No downgrade based on reported CIs (Wang 82.7-88.9%; Shen 85.8-90.3%). No prespecified decision threshold was available.	Not assessable with two studies; this is not evidence that publication bias is absent.	Moderate	One-level downgrade for serious risk-of-bias concern. Descriptive study-specific estimates only; no pooled estimate.
ACR TI-RADS category 5 - specificity	Wang 2026; Shen 2019 (2 studies)	Serious concern; downgrade 1 level. Shen had high risk of bias in patient selection; Wang had unclear risk in patient selection and index test.	No downgrade: estimates were close (90.3% and 87.5%); only two studies, so heterogeneity cannot be estimated reliably.	No downgrade for the narrowly defined category-5 construct; generalizability remains limited.	No downgrade based on reported CIs (Wang 87.8-92.3%; Shen 85.1-89.6%). No prespecified decision threshold was available.	Not assessable with two studies.	Moderate	One-level downgrade for serious risk-of-bias concern. This is not a pooled specificity estimate.
EU-TIRADS category 5 - sensitivity	Wang 2026; Shen 2019 (2 studies)	Serious concern; downgrade 1 level. Shen had high risk of bias in patient selection; Wang had unclear risk in patient selection and index test.	Serious concern; downgrade 1 additional level. Estimates differed (86.3% and 93.3%), with non-overlapping reported 95% CIs; two studies do not permit reliable heterogeneity modelling.	No downgrade for the narrowly defined category-5 construct; external applicability remains limited.	Within-study CIs were reasonably narrow (Wang 82.9-89.1%; Shen 91.3-94.8%), but there is no prespecified decision threshold.	Not assessable with two studies.	Low	Two-level downgrade for serious risk of bias and serious inconsistency. No pooled estimate was calculated.
EU-TIRADS category 5 - specificity	Wang 2026; Shen 2019 (2 studies)	Serious concern; downgrade 1 level. Shen had high risk of bias in patient selection; Wang had unclear risk in patient selection and index test.	No additional downgrade: estimates (85.8% and 80.9%) were directionally similar, although only two studies were available and heterogeneity cannot be estimated reliably.	No downgrade for the narrowly defined category-5 construct; generalizability remains limited.	No downgrade based on reported CIs (Wang 83.1-88.2%; Shen 78.1-83.4%). No prespecified decision threshold was available.	Not assessable with two studies.	Moderate	One-level downgrade for serious risk-of-bias concern. This is not a pooled specificity estimate.

Appendix C 

**Table 8:** Unit of Analysis and Reporting of Multiple Nodules Per Patient.

Study	Patients	Nodules	Diagnostic unit/clustering information	Implication for interpretation
Wang et al. (2026)	1,174	1,174	One nodule per patient in the analyzed cohort. No within-patient multiple-nodule clustering is apparent from these denominators.	The primary 2×2 counts are nodule-level and correspond numerically to the patient denominator.
Shen et al. (2019)	1,568	1,612	More nodules than patients; at least some patients contributed multiple nodules. The available extraction did not establish whether category-specific accuracy estimates or confidence intervals accounted for clustering.	The primary 2×2 counts are nodule-level. Treating all nodules as independent may overstate precision; aggregate 2×2 counts do not permit cluster correction.
Sollmann et al. (2024)	208	322	Multiple nodules per patient. The study is reported to have used generalized estimating equations (GEE) for clustering.	Clustering was addressed in the source analysis; exact category-5 2×2 cells were not available for the primary matched comparison.
Seifert et al. (2021)	849	1,211	Multiple nodules per patient. The available source extraction did not establish whether the reported diagnostic-accuracy estimates accounted for clustering.	Metric-only category-5 results are retained narratively; exact 2×2 cells were not available for the primary analysis.
Słowińska-Klencka et al. (2020)/Jeung and Hong (2022)	866	1,000	One overlapping cohort reported in two publications. The available extraction did not establish whether analyses accounted for within-patient clustering.	Counted as one cohort, not two independent cohorts. Cytology-stratified and companion-report findings are not treated as independent observations.

Appendix D 

**Table 9:** Operational Definitions of the Highest-Risk Categories and FNA Thresholds. ACR: American College of Radiology; TI-RADS: Thyroid Imaging Reporting and Data System; EU-TIRADS: European Thyroid Imaging and Reporting Data System; FNA: fine-needle aspiration

Feature	ACR TI-RADS	EU-TIRADS	Interpretive note
Classification approach	Points are assigned across composition, echogenicity, shape, margins, and echogenic foci; points are summed.	Pattern-based classification into EU-TIRADS 2, 3, 4, or 5.	Different construction rules; categories should not be assumed to represent identical feature thresholds.
Highest-risk category	TR5: total score ≥7 points.	EU-TIRADS 5: at least one high-suspicion feature.	ACR TR5 is a cumulative score; EU-TIRADS 5 is triggered by one or more specified high-risk features.
Features defining highest-risk status	A combination of points from the five feature groups; different combinations can reach ≥7 points.	Non-oval shape, irregular margins, microcalcifications, or marked hypoechogenicity (one or more).	The systems do not map one-to-one at the nodule level.
FNA threshold - highest-risk category	TR5: FNA generally recommended at ≥1.0 cm.	EU-TIRADS 5: FNA generally recommended at ≥10 mm.	The nominal size thresholds are similar, but FNA recommendations remain distinct from category assignment.
Other FNA thresholds	TR3 ≥2.5 cm; TR4 ≥1.5 cm; TR1/TR2 no FNA recommendation.	EU-TIRADS 3 ≥20 mm; EU-TIRADS 4 ≥15 mm; EU-TIRADS 2 generally no FNA.	FNA analyses should not be conflated with the fixed highest-risk-category comparison.

Appendix E 

**Table 10:** PRISMA-DTA Reporting Status Checklist. PRISMA-DTA: Preferred Reporting Items for Systematic Reviews and Meta-Analyses of Diagnostic Test Accuracy Studies; QUADAS-2: Quality Assessment of Diagnostic Accuracy Studies 2; GRADE: Grading of Recommendations Assessment, Development, and Evaluation approach; DOR: diagnostic odds ratio; AUC: area under the curve

PRISMA-DTA domain	Status	Manuscript location/note
Title and identification as a DTA systematic review	Reported	Title/abstract identify the work as a diagnostic accuracy systematic review.
Structured abstract	Updated	Includes search end date, eligibility, author screening/extraction, QUADAS-2, GRADE-DTA, key findings, and synthesis limitations.
Rationale and objectives	Reported	Clinical rationale and primary comparison are described in the Introduction and Objectives.
Eligibility criteria and review question	Reported	Adult population and category-based/FNA-related diagnostic constructs are described in Methods.
Information sources and search dates	Reported with limitations	Six sources searched through October 12, 2026; Embase, Scopus, Web of Science, and Cochrane Library not searched.
Search strategies	Reported	Search concepts and selected strategies are described.
Study selection process	Reported	All three authors participated; disagreements resolved by discussion.
Data collection and data items	Reported	Predesigned Excel extraction and extracted study/population/threshold/reference-standard/accuracy items are described.
Risk of bias and applicability	Reported	QUADAS-2 methods, summary, and figures are included.
Diagnostic accuracy measures	Reported	Sensitivity, specificity, predictive values, likelihood ratios, DOR, and AUC are defined/discussed in the manuscript.
Synthesis methods	Reported	Narrative synthesis; no pooled matched-threshold estimates because only two studies had verified 2×2 data and key heterogeneity/paired data limitations.
Study flow and characteristics	Reported	Counts described: 5,590 identified; 1,457 duplicates; 4,133 screened; 36 sought; 3 not retrieved; 33 assessed; 25 reports/24 cohorts.
Risk-of-bias results	Reported	QUADAS-2 judgments summarized in Results and supplementary figures.
Certainty of evidence	Reported	GRADE-DTA profile supplied as Appendix C.
Discussion and limitations	Updated	Addresses threshold non-equivalence, cohort overlap, clustering, limited databases, lack of registration, and missing retrieval details.
Registration and protocol	Not available	No PROSPERO registration or publicly accessible prospective protocol; do not imply otherwise.
Funding and competing interests	Reported	No external funding; no competing interests declared in the manuscript.
Data availability	Reported	Dataset availability statement included.

Appendix F 

**Table 11:** Search Strategies and Source-Level Search Documentation. ACR: American College of Radiology; TI-RADS: Thyroid Imaging Reporting and Data System; EU-TIRADS: European Thyroid Imaging and Reporting Data System

Source	Search strategy/fields retained in the archive	Records identified
PubMed	("Thyroid Nodule"[Mesh] OR "thyroid nodule"[tiab] OR "thyroid nodules"[tiab] OR "thyroid lesion"[tiab] OR "thyroid lesions"[tiab]) AND ("ACR TI-RADS"[tiab] OR "ACR-TIRADS"[All Fields] OR "American College of Radiology Thyroid Imaging Reporting and Data System"[tiab] OR "American College of Radiology TI-RADS"[tiab] OR "EU-TIRADS"[tiab] OR "EU TI-RADS"[tiab] OR "European Thyroid Association"[tiab] OR "European Thyroid Imaging Reporting and Data System"[tiab] OR "TIRADS"[tiab] OR "TI-RADS"[tiab] OR "Thyroid Imaging Reporting and Data System"[tiab]) AND ("Sensitivity and Specificity"[Mesh] OR "sensitivity"[tiab] OR "specificity"[tiab] OR "diagnostic accuracy"[tiab] OR "diagnostic performance"[tiab] OR "malignancy"[tiab] OR "thyroid cancer"[tiab] OR "thyroid carcinoma"[tiab] OR "comparison"[tiab] OR "comparative"[tiab])	742
Europe PMC	(TITLE_ABS:"thyroid nodule" OR TITLE_ABS:"thyroid nodules" OR TITLE_ABS:"thyroid lesion" OR TITLE_ABS:"thyroid lesions") AND (TITLE_ABS:"ACR TI-RADS" OR TITLE_ABS:"ACR-TIRADS" OR TITLE_ABS:"American College of Radiology TI-RADS" OR TITLE_ABS:"EU-TIRADS" OR TITLE_ABS:"EU TI-RADS" OR TITLE_ABS:"European Thyroid Association" OR TITLE_ABS:"TI-RADS" OR TITLE_ABS:"TIRADS" OR TITLE_ABS:"Thyroid Imaging Reporting and Data System")	1,412
Semantic Scholar via Publish or Perish (PoP)	Four separately run queries are documented: (1) "ACR TI-RADS" thyroid nodules diagnostic accuracy; (2) "EU-TIRADS" thyroid nodules diagnostic accuracy; (3) ACR TI-RADS EU-TIRADS comparative diagnostic performance thyroid; (4) a broader query described in the archive as combining thyroid imaging reporting data system / thyroid ultrasound risk stratification, ACR or European systems, and nodule malignancy. The exact text of query 4 and PoP export/settings metadata are not retained.	224
Google Scholar via PoP	Four separately run queries are documented: (1) "ACR TI-RADS" thyroid nodules diagnostic accuracy; (2) "EU-TIRADS" thyroid nodules diagnostic accuracy; (3) ACR TI-RADS EU-TIRADS comparative diagnostic performance thyroid; (4) a broader query described in the archive as combining thyroid imaging reporting data system / thyroid ultrasound risk stratification, ACR or European systems, and nodule malignancy. The exact text of query 4 and PoP export/settings metadata are not retained.	55
OpenAlex via PoP	Four separately run queries are documented: (1) ACR TI-RADS thyroid nodules diagnostic accuracy; (2) EU-TIRADS thyroid nodules diagnostic accuracy; (3) ACR TI-RADS EU-TIRADS comparative thyroid nodules; (4) thyroid ultrasound risk stratification systems malignancy ACR European. The archive does not retain individual query timestamps or complete PoP settings.	374
Crossref via PoP	Four separately run queries are documented: (1) ACR TI-RADS thyroid nodules; (2) EU-TIRADS thyroid nodules; (3) ACR TI-RADS EU-TIRADS; (4) Comparative diagnostic accuracy of thyroid nodule risk stratification systems. The archive does not retain individual query timestamps or complete PoP settings.	2,783

Appendix G 

**Table 12:** Operational Eligibility Criteria and Handling of Study Types. ACR: American College of Radiology; TI-RADS: Thyroid Imaging Reporting and Data System; EU-TIRADS: European Thyroid Imaging and Reporting Data System; FNA: fine-needle aspiration; QUADAS-2: Quality Assessment of Diagnostic Accuracy Studies 2; CAD: computer-aided diagnosis; TP: true positive; FP: false positive; FN: false negative; TN: true negative

Domain	Eligibility/handling rule	Application in this review
Population	Adult thyroid-nodule population; adults defined as age ≥18 years.	Mixed-age reports were eligible only if adult data could be separated. One report was excluded because adult data could not be separated from participants <18 years.
Index test	ACR TI-RADS and/or EU-TIRADS.	A direct head-to-head comparison was not required for inclusion in the descriptive review. Single-system reports could contribute relevant context.
Target condition	Malignancy versus benign thyroid nodule, as defined in each included report.	The target outcome and analytic denominator were extracted as reported; differences were not silently harmonized.
Diagnostic endpoint	Fixed category threshold, FNA recommendation/biopsy rule, or another explicitly defined risk-stratification comparison.	FNA-decision outcomes, fixed category thresholds, subgroup analyses, and alternative thresholds were kept separate. The primary comparison required ACR TR5 versus EU-TIRADS 5.
Reference standard	Reference standards reported by the original study were recorded; the included set used histopathology, cytology/FNA, mixed cytology/histopathology, or clinical/imaging follow-up.	No single uniform reference standard was imposed in the documented eligibility record. Differences and verification pathways informed QUADAS-2 and interpretation.
Study design	Prospective and retrospective clinical diagnostic-performance studies were retained.	No exclusion based solely on prospective versus retrospective design is documented. The archive does not support claiming a more restrictive design rule.
CAD/modified systems	Relevant CAD-assisted or modified-system reports could be retained for descriptive synthesis.	CAD findings were kept distinct from routine-reader classification and were not pooled with the primary matched category-5 estimates.
Selected populations/subgroups	Selected surgical/biopsy cohorts and cytology, small-nodule, or disease-specific subgroups could be retained where relevant.	Selection and spectrum limitations were described; subgroup-specific estimates were not treated as population-wide or interchangeable.
Exact 2×2 data	Exact TP, FP, FN, and TN counts required for recalculated primary quantitative comparisons.	Counts were not reconstructed from rounded sensitivity/specificity alone. Only two studies contributed verified exact 2×2 data to the primary matched category-5 comparison.
Overlapping reports	Companion reports could be retained for relevant additional information but not counted as independent cohorts.	Jeung and Hong (2022) was treated as an overlapping/companion report of Słowińska-Klencka et al. (2020).
Exclusion at final reconciliation	Exclude a mixed-age report when adult-only results cannot be separated.	One provisionally retained report was excluded for this reason.
Unrecorded protocol details	Do not infer undocumented language, publication-type, or additional design/reference-standard restrictions.	The available archive does not preserve a complete prospective protocol specifying every such rule.

Appendix H 

**Table 13:** Previous Systematic Reviews and Meta-Analyses Relevant to ACR TI-RADS and EU-TIRADS. ACR: American College of Radiology; EU-TIRADS: European Thyroid Imaging and Reporting Data System; ATA: American Thyroid Association; K-TIRADS: Korean Thyroid Imaging Reporting and Data System; TI-RADS: Thyroid Imaging Reporting and Data System; C-TIRADS: Chinese Thyroid Imaging Reporting and Data System; AUC: area under the curve

Review	Evidence base/question	Main findings relevant to this review	Relationship to the present review
Kim PH et al., 2020 [40]	29 studies; 33,748 nodules; category-based diagnostic accuracy across ACR, EU, ATA, and K-TIRADS.	At category 5, pooled sensitivity/specificity: ACR 66%/91%; EU 82%/90%. Overall system performance was considered comparable; heterogeneity was substantial.	Indirect category-based benchmark. Not a paired analysis restricted to the same nodules or exact common feature threshold.
Kim DH et al., 2020 [35]	34 studies; 37,585 nodules; accuracy at category 5 and category 4/5.	For category 5, EU-TIRADS sensitivity 78% versus ACR 70%; specificity 89% for both. Differences were not statistically significant.	Supports a descriptive trend toward greater EU sensitivity, but pooled estimates combine heterogeneous studies and are not a within-cohort paired comparison.
Li et al., 2021 [36]	16 studies; 18,614 patients; 21,882 nodules; ACR TI-RADS diagnostic performance.	Pooled ACR sensitivity 89%, specificity 70%; estimates span the thresholds included in that review.	ACR-only benchmark; does not provide a direct ACR-versus-EU comparison at category 5.
Piticchio et al., 2024 [37]	7 studies; 41,332 nodules; head-to-head comparison of ACR-, EU-, and K-TIRADS category distributions and assigned malignancy risks.	Found differences in low-risk category distribution and category-specific malignancy-risk calibration; principal outcomes were not pooled category-5 sensitivity/specificity.	Directly compares classification systems but addresses category allocation/risk calibration rather than diagnostic accuracy at the exact primary threshold in this review.
Jin et al., 2023 [38]	Single retrospective cohort; 3,438 nodules in 3,013 patients; C-TIRADS, ACR TI-RADS, and EU-TIRADS.	ACR sensitivity 89.1%, specificity 69.5%; EU sensitivity 78.4%, specificity 78.9%; AUC 0.844 and 0.802, respectively.	Direct cohort comparison, but broader classification/management definitions are not equivalent to the restricted matched category-5 analysis.
